# Neuroprosthetic closed-loop strategy for sustained blood pressure reduction via simultaneous stimulation and recording from the spinal cord

**DOI:** 10.1016/j.neurot.2025.e00758

**Published:** 2025-09-30

**Authors:** Mingeun Cho, Minhye Choo, Matthew Koh, Sunguk Hong, Junseung Mun, Juho Koo, ChangHwa Oh, Sung-Min Park

**Affiliations:** a Department of Mechanical Engineering, Pohang University of Science and Technology (POSTECH), Pohang, South Korea; b Department of Convergence IT Engineering, Pohang University of Science and Technology (POSTECH), Pohang, South Korea; c Department of Medical Science and Engineering, Pohang University of Science and Technology (POSTECH), Pohang, South Korea; d Biomedical Engineering Research Center, Samsung Medical Center, Seoul, 06351, South Korea; e Department of Electrical Engineering, Pohang University of Science and Technology (POSTECH), Pohang, South Korea; f Division of Interdisciplinary Bioscience and Bioengineering, Pohang University of Science and Technology, Pohang, 37673, South Korea

**Keywords:** Closed-loop, Neuromodulation, Hypertension, Spinal cord stimulation

## Abstract

Sympathetic hyperactivity is a common feature observed in both primary and secondary hypertension, and neuromodulation-based therapies targeting this overactivity are under active investigation. The intermediolateral nucleus (IML) in the spinal cord plays a central role in sympathetic regulation, and its direct stimulation induces a transient, frequency-dependent reduction in blood pressure. However, this effect gradually diminishes as the baroreflex restores blood pressure to baseline. Therefore, this study aims to develop a closed-loop (CL) stimulation system that adjusts parameters in real-time based on neural activity recorded from the IML and to enhance and prolong the transient antihypertensive effect. This strategy was evaluated in normotensive and angiotensin II-induced hypertensive rat models and compared to open-loop (OL) stimulation of equal intensity. In both models, CL stimulation produced a more sustained blood pressure reduction than that of OL stimulation. These findings suggest that CL spinal cord stimulation offers a more effective and durable therapeutic option for treating hypertension.

## Introduction

Hypertension affects approximately 1.3 billion people globally and causes around 8.5 million deaths annually [1,2]. Although the causes of hypertension are diverse, sympathetic overactivity is frequently observed in individuals with high blood pressure, suggesting that autonomic nervous system imbalance may play a fundamental role in the development of the condition [[3], [4], [5]]. Pharmacotherapy is the current standard treatment for hypertension, but it often requires multiple drugs to achieve and maintain adequate blood pressure control [6,7]. Despite growing interest in conditions such as resistant hypertension, the effectiveness of pharmacological treatment remains limited owing to the challenges associated with polypharmacy, including adverse effects and poor medication adherence [[8], [9], [10], [11]]. Currently, conventional therapies enable successful blood pressure control in only a quarter of patients with hypertension, underscoring the urgent need to develop alternative treatment strategies beyond pharmacological interventions [1].

Neuromodulation has recently emerged as a sustained therapeutic approach for hypertension by targeting the overactivity of the sympathetic nervous system [[12], [13], [14]]. These techniques have primarily been employed as adjunctive strategies to enhance blood pressure reduction in patients with treatment-resistant hypertension alongside pharmacological therapy [15,16]. Furthermore, previous studies have proposed the potential of neuromodulation as a stand-alone treatment for individuals who respond poorly to medication or face challenges with adherence to pharmacological regimens [17]. Although the brain and baroreceptors have traditionally been the primary targets for stimulation [15,18,19], concerns about unpredictable side effects, limited effectiveness, and restricted surgical accessibility have spurred interest in alternative sites [20,21]. To enhance the therapeutic potential of such interventions, it is crucial to identify stimulation targets that centrally regulate sympathetic activity while minimizing invasiveness and the risk of adverse outcomes [14].

The spinal cord functions as a central hub for blood pressure regulation by relaying descending sympathetic signals from the brainstem to peripheral organs and mediating spinal reflexes [22,23]. In particular, the intermediolateral nucleus (IML) is a critical structure for sympathetic regulation within the spinal cord, comprising sympathetic preganglionic neurons (SPNs) and inhibitory interneurons that modulate SPN activity [24,25]. Owing to this neuronal composition, the IML functions as a principal regulatory site for relaying sympathetic output from the central nervous system to peripheral organs. [23,26,27]. Stimulation of brainstem regions involved in blood pressure regulation, such as the rostral ventrolateral medulla (RVLM) and nucleus tractus solitarius (NTS), elicits changes in IML neural activity [[28], [29], [30]]. Furthermore, epidural spinal cord stimulation (eSCS) demonstrates the ability to modulate sympathetic output via the IML pathway [23,31]. These findings indicate that the spinal cord—particularly the IML—represents a promising neuromodulation target for hypertension treatment.

**Fig. 1:**
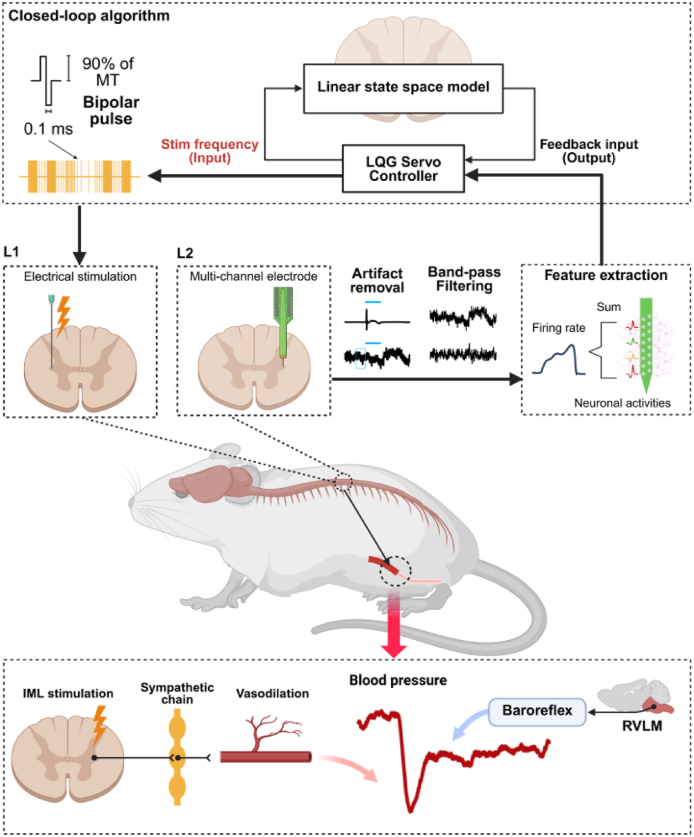
**Schematic representation of the closed-loop stimulation strategy.** Signals recorded at the lumbar level 2 of the spinal cord via a multichannel electrode are processed for artifact removal and filtering, then summed and used as feedback input for the control system. The closed-loop algorithm computes the optimal stimulation frequency, which is delivered to the at the lumbar level 1 of the spinal cord intermediolateral nucleus. Spinal cord stimulation reduces blood pressure via sympathetic inhibition and vasodilation, followed by gradual recovery due to baroreflex competition. Abbreviations: IML, intermediolateral nucleus; RVLM, rostral ventrolateral medulla.

Previous studies on blood pressure regulation via neuromodulation have primarily used open-loop (OL) stimulation, which applies fixed, predefined stimulation parameters [15,17,[32], [33], [34], [35]]. However, because blood pressure is regulated by physiological homeostatic mechanisms [36], OL stimulation cannot adjust to these dynamic changes, making it difficult to sustain stable blood pressure reduction over time. Numerous studies report that blood pressure lowered by OL stimulation often returns to baseline—not only after stimulation stops but also during continuous stimulation—indicating the effect is temporary and unable to maintain stable reduction [37,38]. This response may be interpreted as the baroreflex perceiving the decrease in blood pressure as a disruption of homeostatic and initiating compensatory mechanisms to restore it to baseline levels [39]. Therefore, this study aims to address these limitations by developing a closed-loop (CL) stimulation strategy that utilizes physiological feedback to automatically adjust stimulation intensity, thereby prolonging the duration of blood pressure reduction. To this end, a computational model was developed to simulate the neural dynamics of the IML, which regulates sympathetic output. Based on this model, an algorithm was designed to dynamically modulate IML neural activity by adjusting the stimulation frequency (Fig. 1). This CL algorithm operates independently of supraspinal centers and functions as a neuroprosthetic neural circuit, enabling rapid responses analogous to spinal reflexes. The present study highlights neuromodulation targeting the spinal cord as a fundamental therapeutic strategy for hypertension and underscores the potential of the CL strategy in enhancing the effectiveness of neuromodulation-based blood pressure control.

## Materials and Methods

### Animals

Adult male Sprague–Dawley rats (age: 9–12 weeks old; weight: 280–350 ​g; supplied by Orient Bio Inc., Seongnam, Republic of Korea) were used in the experiments. All procedures were approved by the Institution of Animal Care and Use Committee of the Pohang University of Science and Technology (POSTECH-2023-0094) and conducted in accordance with National Institutes of Health guidelines. At the conclusion of the experiments, the rats were euthanized using CO_2_ inhalation.

### Surgery procedure

Inhalation anesthesia was induced in a rat using isoflurane (5 ​% in O2 at a flow rate of 0.4–0.6 ​l/min; Piramal Critical Care Inc., Pennsylvania, USA), followed by intraperitoneal (IP) administration of urethane (1.4 ​g/kg, 0.2 ​g/ml; Sigma Aldrich). Supplemental doses of urethane were administered as necessary to maintain anesthesia throughout the experimental period, which lasted 4–7 ​h. Tracheal intubation was performed to secure the airway. Continuous arterial pressure monitoring was achieved via femoral artery catheterization (SP-10, Natsume Seisakusho Co. Tokyo, Japan) (Fig. S1A). After catheterization, the rat was placed in a stereotaxic frame (RWD Life Science, Guangdong, China), and a laminectomy was performed at the T11–T12 spine. Following the laminectomy, the spinal cord was stabilized using a spinal cord adaptor (Biomed 30–2026, Guangdong, China) by securing the T10 and T13 vertebrae to minimize motion artifacts caused by respiration and body movements.

Electrodes were inserted into the spinal cord for stimulation and neural signal measurement (Fig. S1B). Spinal cord stimulation was performed using a bipolar configuration consisting of two insulated stainless steel wires (polyester-coated AISI 316 stainless steel wire, diameter: 150 ​μm; GoodFellow). The wires were coated with polyester insulation to prevent short circuits, with only the tips exposed for stimulation. Both electrodes were inserted approximately 1.0–1.2 ​mm into the left ventral region of the spinal cord at the T13 level (Fig. S2A).

To record neural activity from the IML, a needle-shaped multichannel probe (A1x16-Poly2s-5mm-50s-177-A16; NeuroNexus Technologies, Michigan, USA) was inserted approximately 1–1.2 ​mm into the left ventral region of the spinal cord at the L1 IML level. Ground and reference electrodes (stainless steel, 200 ​μm diameter; GoodFellow, Cambridge, UK) (Fig. S2B) were inserted into the right side at the T13 spinal cord level. Electrode positions were carefully adjusted within a 0.3 ​mm range to optimize action potential detection.

### Stimulation design and configuration

Electrical stimulation was delivered using a commercial stimulator (IZ2-32, 32-channel stimulator; Tucker–Davis Technologies, Florida, USA). Because motor neurons are located near the IML, adjusting stimulation amplitude and frequency simultaneously often triggered adverse side effects—such as elevated blood pressure or tremors exceeding the motor threshold—which compromised the accuracy of data interpretation. To ensure safety and maintain precise control over stimulation, only the frequency was varied during the experiment. Before the experiment, the motor threshold was identified by gradually increasing the current until a tremor was observed. Stimulation was then applied at up to 90 ​% of this motor threshold to minimize tremor-induced interference (amplitude range: 90–200 ​μA). The pulse width was fixed at 100 μs.

### Neural data recording and post-processing

Recorded signals were amplified using a commercial amplifier (PZ5 Neurodigitizer Amplifier; Tucker-Davis Technologies) and processed with a bioamp processor (RZ2 Bioamp Processor; Tucker-Davis Technologies). The processor was connected to a PC and operated using MATLAB 2015b (MathWorks, Massachusetts, USA). During recordings, action potentials were detected using a threshold set at 5.5 times the root mean square value over a 5-s window in OpenProject. (Tucker-Davis Technologies) To ensure accurate calculation of the firing rate, stimulation-induced artifacts were removed through post-processing using interpolation in MATLAB (Supplementary Note 1) (Fig. S3). The firing rate was calculated using the Tucker-Davis Technologies (TDT) software development kit and defined as the number of action potentials per second. For 3 ​s after the start of the recording, the signal was not properly measured due to the characteristics of the TDT equipment, which was excluded from the signal processing process.

Owing to the size of the electrode relative to the IML, spikes were not detected on all channels. Additionally, processing high-sampling-rate signals from all channels for spike detection would require considerable computational resources. To address these limitations, only channels with detectable spikes were analyzed. The firing rates from four selected channels were summed and used as a feature input for the feedback system. To identify appropriate channels for analysis, a 20 ​Hz stimulation was applied prior to the main experiment. The stimulation intensity was set at the motor threshold or at a level that produced an approximately 10 ​mmHg reduction in blood pressure. Subsequently, the temporal firing rate responses of each channel were analyzed offline. Four channels exhibiting increased firing rates during continuous stimulation were selected for integration into the feedback system.

### Recording and preprocessing blood pressure

Arterial pressure was recorded using a commercial multi-channel system (IX-RA_834, iWorx, New Hampshire, USA) equipped with a pressure transducer (BP-102, iWorx) and operated through proprietary LabScribe software (iWorx). To minimize hydrostatic artifacts, the pressure transducer was placed at the heart level. Data were sampled at 100 ​Hz, and key events, such as the onset of stimulation, were time-stamped. Blood pressure data were exported in “.mat” format and analyzed in MATLAB 2022b (MathWorks Inc.). To calculate mean arterial pressure (MAP), a 2nd-order Butterworth band-pass finite impulse response filter with a 0.05–0.5 ​Hz cutoff frequency was applied. For evaluating changes in blood pressure, baseline variability was controlled by using the average blood pressure measured during the 30 ​s preceding stimulation as the reference.

**Fig. 2:**
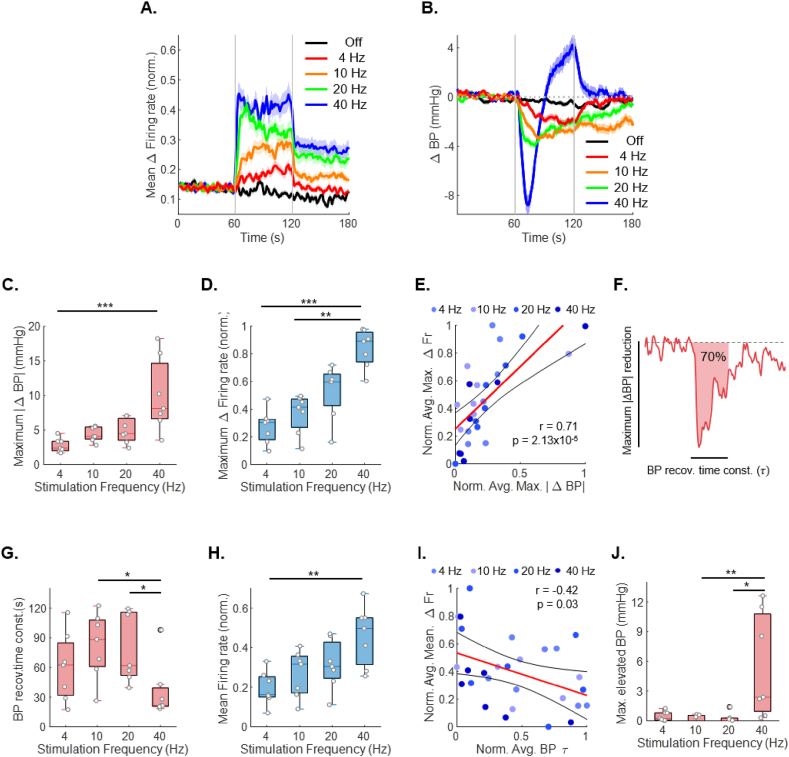
**Parametric assessment of frequency-induced neuronal and hemodynamic responses in intermediolateral nucleus stimulation. A.** Averaged, normalized IML firing rate response for each stimulation frequency. Firing rates were normalized for average calculation. (*n* ​= ​7) **B.** Average reduction in blood pressure for each stimulation frequency. **C.** Maximum blood pressure reduction increased with stimulation frequency. **D.** Maximum change in IML firing rate increased with stimulation frequency. **E.** A significant linear correlation was observed between normalized average maximum firing rate and blood pressure reduction, suggesting a frequency-dependent relationship between neural and hemodynamic responses. **F.** Evaluation metric for assessing the blood pressure reduction effect. The blood pressure recovery time constant was defined as the time required to reach 70 ​% of the recovery following the blood pressure reduction. **G.** The time constant of blood pressure recovery decreased with increasing frequency. **H.** Mean IML firing rate at each stimulation frequency. **I.** Linear correlation between normalized average mean firing rate and blood pressure recovery time constant. **J.** Maximum increase in blood pressure above baseline during stimulation. For panels A and F, data are shown over a 60-s window before and after stimulation. The mean values are plotted as lines, and the shaded areas represent the 95 ​% confidence intervals. For panels C, D, G, H and J data are presented as boxplots (median, IQR, and 1.5 ​× ​IQR whiskers) with individual data points. Group differences were assessed using the Kruskal–Wallis test with Dunn–Šidák post hoc correction. Significant pairwise differences are denoted by asterisks (n.s., not significant; ∗*p* ​< ​0.05, ∗∗*p* ​< ​0.01, ∗∗∗*p* ​< ​0.001). Panel E and I reports Pearson’s correlation coefficient. Each colored dot represents the result of an experiment conducted at the same stimulation frequency. The red line indicates the fitted linear regression line, and the black curves denote the 95 ​% confidence interval. Abbreviations: OL, open-loop; CL, closed-loop.

To quantify blood pressure recovery, the “blood pressure recovery time constant” was defined as the time required for pressure to return to 70 ​% of the baseline following its minimum value (Fig. 2F). Prior to calculating the time constant, the blood pressure signal was filtered using a second-order low-pass Butterworth filter with a 2 ​Hz cutoff to remove high-frequency noise while preserving the overall trend of the signal. The recovery time constant was then determined as the time point at which the filtered signal first crossed the 70 ​% baseline threshold.

### Exclusion criteria and data quality control

Following surgery, blood pressure was measured. If it was determined to be excessively low, the experiment was deemed unfeasible, and the animal was euthanized. If blood pressure measurement became impossible due to catheter occlusion caused by thrombus formation over time, additional heparin was administered to dissolve the clot. If blood pressure remained unmeasurable despite these efforts, all subsequent data were excluded from analysis. Experiments were conducted in a space isolated from external vibration. However, if neural signals were contaminated by external factors such as unintended vibration, electrostatic interference, or electromagnetic noise from nearby equipment, the corresponding trial was excluded from the analysis.

Even when identical stimulation patterns are applied across trials, the resulting neural responses are substantially influence by intrinsic neural dynamics that fluctuate within the same subject [40]. Previous studies show that, even with consistent targeting and stimulation parameters, the evoked neural responses in the same subject vary in magnitude and pattern across trials. This variability likely reflects fluctuations in the intrinsic state of the nervous system at the time of stimulation [41,42]. To distinguish stimulation effects from intrinsic variability, trial-level statistical analysis is essential, as it provides a robust means of capturing the complexity of neural responses that simple averages cannot adequately represent [43,44]. Consistent with this perspective, trial-level statistical analyses were conducted for both firing rate, which reflects neural activity, and blood pressure–related parameters that are functionally associated with neural activation. These analyses were performed in parallel with animal-level statistical comparisons. Details of all statistical analyses can be found in the supplementary materials (Supplementary Table 1).

### Parametric stimulation to investigate the temporal relationship between intermediolateral nucleus activity and blood pressure

Given its critical role in regulating sympathetic output and blood pressure, neuronal activity within the IML was hypothesized to serve as a feedback variable for the CL stimulation algorithm. To validate this hypothesis, stimulation frequency was systematically varied, and the corresponding neural and blood pressure were analyzed. Neural and hemodynamic responses were recorded during electrical stimulation at four frequencies (4, 10, 20, and 40 ​Hz), with each lasting 60 ​s. A 300-s rest period between sessions was provided to ensure adequate physiological recovery. The stimulation current was set below the motor threshold to elicit a hemodynamic response without inducing motor activation, while all parameters except frequency and stimulation duration were kept constant. The stimulation protocol comprised one set of four frequencies, repeated twice. Neural and blood pressure signals were post-processed and analyzed using MATLAB.

### Designing data-driven input-output model and linear quadratic Gaussian controller

In a CL algorithm, an accurate model is essential for predicting input-output relationships and designing an effective controller. The model directly influences the overall stability, responsiveness, and therapeutic efficacy of the system. Leveraging the previously established correlation between IML neural activity and blood pressure, a data-driven linear state-space model (LSSM) was developed to utilize IML activity as a feedback variable in the CL algorithm [42]. This model was designed to simulate the dynamic characteristics of IML neural activity and predict neural responses to changes in stimulation frequency. Given that the behavior of the model could be adequately simulated using low-dimensional data [45] and to optimize training speed and enable real-time computation, the model was trained using four dimensions.

From a neurodynamic perspective, each subject exhibits intrinsic and stimulus-evoked response dynamics. As the number of trials increases and more data accumulate, the influence of intrinsic dynamics on the learning process diminishes, while the contribution of stimulus-driven response dynamics becomes more pronounced. To minimize the effect of intrinsic dynamics, repeated trials with identical stimulation patterns were used for model training. To enable efficient model learning without direct experimentation across various stimulation conditions, a 180-s binary stochastic stimulation pattern with white Gaussian noise characteristics served as input to elicit neural responses across a wide frequency range (Fig. S4A).

For each subject, ten 180-s datasets were collected, and the baseline offset was removed by subtracting the mean. The data were divided into 135-s training sets and 45-s test sets for 4-fold cross-validation. The training sets were concatenated to construct the four-dimensional LSSM (Fig. S4B). To further minimize the influence of intrinsic dynamics in the test sets, the ground-truth input-driven dynamics were obtained by averaging repeated trials, thereby isolating the stimulus-induced component of the neural response. When the training input was supplied to the trained model for forward prediction, the ground-truth input-driven dynamics were accurately reproduced, demonstrating that the input–output dynamics of the IML neural network were successfully captured (CC ​= ​0.9517; Fig. S4C).

(3) $$x_{t+1}=Ax_{t}+Bu_{t}+w_{t}\text{,}$$

(4) $$y_{t}=Cx_{t}+Du_{t}+v_{t}\text{,}$$

A Linear Quadratic Gaussian (LQG) controller was developed based on the constructed LSSM. By integrating a Linear Quadratic Integrator with a Kalman filter, the LQG controller achieves optimal performance under conditions of noise and model uncertainty. The incorporation of a Kalman filter enables effective operation in uncertain environments while ensuring robustness. Furthermore, its low computational complexity makes it well-suited for real-time control applications and deployment on embedded devices with hardware limitations.

(5) $$J=\sum_{t=0}^{\infty }z_{t}^{T}Qz_{t}+{\left(u_{t}-u_{r}\right)}^{T}R\left(u_{t}-u_{i}\right)$$

$z_{t}={\left[x_{t}^{T}\;e_{t}\right]}^{T}\text{,}$ where $u_{i}$ is the initial input parameter, and $e_{t}$ represents the difference between the reference r and IML firing rate $y_{t}$, and it regulates the rate of input change in a manner similar to the gain factor used in classical PID control.

(6) $$e_{t}=T_{s}\sum_{k=0}^{t}\left(r-y_{t}\right)$$

(7) $$u_{t}=-\left[K_{x}\;K_{i}\right]\left[\begin{array}{c} x_{t}\\ e_{t} \end{array}\right]$$

$K_{x}$ is the feedback gain for the original state $x_{t}$, and $K_{i}$ is the feedback gain for the ${\;e}_{t}$. $T_{s}$ is the unit time step of a discrete-time system. In this experiment, we set $T_{s}$ as 1 ​s. By adjusting Q and R, which represent the weighting matrices for the state and input, the speed of input change can be regulated. The Kalman filter was used to estimate the hidden state to minimize the discrepancy between the model output and values obtained from *in vivo* experiments. The Kalman filter equations are expressed as follows.

(7) $${\hat{x}}_{t+1|t}=A{\hat{x}}_{t|t}+Bu_{t}$$

(8) $${\hat{x}}_{t+1|t+1}={\hat{x}}_{t+1|t}+L\left(y_{t+1}-C{\hat{x}}_{t+1|t}\right)$$

${\hat{x}}_{t+1|t}$ denotes the predicted next state based on the current available information. The Kalman gain matrix *L* is computed using the MATLAB function “kalman,” which accounts for noise in the input and measurement processes.

### *In vivo* validation of the closed-loop feedback stimulation strategy

A CL stimulation system was developed based on the previously designed LQG controller. Results from preceding experiments show that low-frequency stimulation fails to induce a sufficient reduction in blood pressure. In contrast, high-frequency stimulation produces a more pronounced transient decrease in blood pressure but also accelerates recovery to baseline by increasing the mean firing rate of the IML, which is directly associated with sympathetic outflow. These findings highlight the necessity of optimizing stimulation frequency. Accordingly, the objective of CL is to continuously modulate stimulation frequency within an optimal range to maximally suppress increases in IML neural firing rate, and thereby sustain a robust blood pressure-reducing effect over an extended duration.

The performance of the CL stimulation strategy was assessed through *in vivo* experiments conducted in normotensive rats. Raw neural signals were processed in real-time using the TDT system, where band-pass filtering and stimulation artifact removal were performed. Only the detected spikes were automatically transferred to MATLAB, where the LQG algorithm was operated. This algorithm utilized the summed firing rate from four selected channels of the 16-channel array as the feedback variable.

The stimulation frequency was adjusted in real time based on a gain factor that scaled the difference between the predefined reference value and the measured firing rate, thereby determining the direction and magnitude of input adjustment. The primary purpose of this continuous frequency modulation was not to fix the feedback variable at a specific value, but rather to maximize the blood pressure-reducing effect of stimulation by persistently varying the stimulation intensity to counteract recovery phenomena arising from compensatory mechanisms [46,47]. To mitigate the attenuation of stimulation effects caused by neural adaptation and to reinforce the durability of the blood pressure-reducing effect, an upward-ramping input pattern was implemented in which the stimulation intensity was steadily increased by adjusting the input weighting [48]. The input was constrained to remain below a predefined upper limit, enabling effective artifact removal and minimizing side effects such as tremors. This approach enabled more stable and adaptive blood pressure control by preventing abrupt elevations in blood pressure. The system operates autonomously, independent of higher brain centers, and is designed to mimic a reflex-like neural circuit capable of rapid, spinal-level responses.

To verify the feasibility of the proposed CL stimulation compared to that of the OL stimulation, the stimulation duration was set to 180-s, and a 600-s rest period was implemented between the stimulations to allow recovery of blood pressure and neural activity. The initial frequency was set at 11 ​Hz. To reduce side effects such as tremors and minimize signal distortion caused by imperfect artifact removal, the stimulation frequency was capped at 30 ​Hz. This constraint was implemented to prevent the CL input from rapidly reaching its upper limit and subsequently operating in the same manner as OL stimulation. This measure ensures a clear distinction between the two stimulation modes and enables precise evaluation of their differential effects. Since the reference value serves as a key parameter for the LQG controller in determining the rate and direction of input change relative to the actual output, it was optimized to provide a sufficient rate of input increase for clear differentiation from OL while preventing excessive escalation. In actual CL experiments, a decrease in the mean firing rate of the IML was observed during sustained stimulation. Accordingly, the reference value was set above baseline to ensure that the gain factor remains elevated, thereby maintaining and reinforcing the upward-ramping input pattern and enhancing the blood pressure-reducing effect. In simulations, setting the reference of 40 units above baseline ensures that the stimulation frequency remains stably below 30 ​Hz throughout the entire 3-min stimulation period. However, unlike the generalized simulation model, actual experiments are subject to intrinsic neural dynamics that may produce variable response patterns. Accordingly, in this study, the reference value was conservatively set within a range of 20–40 units above baseline, adjusted according to individual neural responses. Following CL stimulation, the average stimulation frequency from each set was applied to the OL protocol to minimize differences in stimulation intensity and ensure a fair comparison. To maintain high data quality, each stimulation condition was repeated across three sessions.

### Analysis of the relationship between intermediolateral neural firing and blood pressure

After completing the experiments, spike sorting was performed using the open-source software “Klusta” to evaluate how CL stimulation affected individual neuronal activity. To determine whether neurons in the IML encoded blood pressure-related information, mutual information (MI) was computed between the firing rate of each neuron and blood pressure during the pre-stimulation, stimulation, and post-stimulation periods. MI is an information-theoretic measure that quantifies the shared information between two probabilistic variables, capturing linear and nonlinear dependencies.

To assess the statistical significance of the MI value for each neuron, a nonparametric, shuffle-based permutation test was performed. Blood pressure time-series data were randomly shuffled 100 times, and the MI between each shuffled sequence and the corresponding firing rate was recalculated to regenerate a null distribution. The p-value was determined based on the position of the observed MI value within the null distribution. Neurons with *p*-values <0.05 were considered to convey statistically significant information regarding blood pressure.

Subsequently, to determine whether the firing rate used as the feedback input in this study adequately reflected information about individual neurons and blood pressure, linear regression models were employed to predict neural activity and blood pressure. By comparing the predicted and actual values and calculating their correlation coefficients, the extent to which the firing activity of individual neurons accounts for variations in blood pressure and aggregate neural signals was evaluated. Finally, individual neurons were classified as excitatory or inhibitory based on their responses to stimulation, and the average responses were examined. Neurons were classified as excitatory if their activity increased from baseline after stimulation and inhibitory if their activity decreased. This classification, reflecting the characteristic composition of the IML—which contains excitatory and inhibitory neurons—was performed to analyze how each neuronal population responded to stimulation, contributing to the resulting hemodynamic changes.

### Implement of closed-loop feedback strategy in angiotensin-II induced hypertensive model

To evaluate the therapeutic potential of the CL stimulation strategy for hypertension, its acute effects were tested in rats with Angiotensin II-induced hypertensive model. The Angiotensin II-induced hypertension model was chosen because it effectively simulates hypertension-induced cardiovascular remodeling within a short period. (Supplementary Note 2) (Fig. S5).

An osmotic pump (Model 2004, ALZET LLC., California, USA) filled with Angiotensin-II (human; A9525, Sigma Aldrich) was implanted subcutaneously into male Sprague–Dawley rats (age: 8 weeks; weight: 200–250 ​g; Orient Bio Inc) for 4 weeks. The infusion rate (0.7 ​mg/kg/day) was determined considering the dose conversion rate between rats and mice [49] and was selected to induce cardiac hypertrophy and vascular fibrosis [50,51].

Blood pressure and body weight were measured using the tail-cuff method every 3–5 days at 10 a.m., prior to the experimental procedure. The measurements were performed under inhalation anesthesia with 1 ​% isoflurane, utilizing the IITC Blood Pressure Measurement System (1R229, IITC Life Science Inc., California, USA). Considering previous findings that indicate a gradual decline in the osmotic pump infusion rate over time, the experiment was conducted during the 4th week after implantation [52].

Given the hypertensive condition and blood pressure response to stimulation, the maximum stimulation frequency was set to 30 or 40 ​Hz to optimize the blood pressure-lowering effect. Aside from this adjustment, all other experimental protocols were consistent with those used in the normotensive animal models. After the experiment, histological examinations of the heart and both kidneys were performed to confirm the presence of hypertension. Hematoxylin & Eosin (H&E) staining was used to evaluate tissue structure and pathological changes, with particular emphasis on vascular remodeling, inflammatory responses, and fibrosis.

## Result

### Direct intermediolateral nucleus stimulation mechanisms lowering blood pressure

The IML is widely recognized as a sympathetic excitatory center, densely populated with sympathetic preganglionic neurons [53,54]. Given its central role in blood pressure regulation, we investigated whether direct electrical stimulation of the IML could reliably induce a reduction in blood pressure.

To investigate the mechanism by which direct stimulation of the IML reduces blood pressure, guanethidine—a preganglionic sympathetic blocker—was administered. Before guanethidine injection, IML stimulation elicited a clear blood pressure reduction. However, following administration, the blood pressure response was completely abolished (Fig. S6A). Notably, neural activity in the IML remained unchanged, consistent with the pharmacological action of intraperitoneally administered guanethidine, which inhibits peripheral sympathetic transmission without affecting neural firing within the sympathetic chain. These results show that the blood pressure-lowering effect of IML stimulation is mediated by inhibiting sympathetic activity.

Meanwhile, since parasympathetic activation can also lead to blood pressure reduction, atropine, a parasympathetic blocker, was administered to assess its effect on IML stimulation. As a result, although the blood pressure-lowering response and the increase in neural activity were slightly reduced after atropine administration, they were still observed (Fig. S6B). These results align with that of previous chemical ablation studies employing various autonomic blockers to investigate the mechanism of eSCS [31]. Therefore, direct stimulation of the IML likely operates via a mechanism similar to that of eSCS, involving the inhibition of sympathetic activity and vasodilation mediated by the release of calcitonin gene-related peptide (CGRP), ultimately resulting in a reduction in blood pressure [55]. Additionally, to determine whether direct stimulation of the IML modulates blood pressure through the activation of supraspinal circuits, c-Fos expression was examined in the NTS, a key center of parasympathetic nervous system regulation connected to the vagus nerve (Fig. S7) [56,57]. No c-Fos activation was observed, indicating that spinal cord stimulation regulates blood pressure via local spinal circuits without involving higher brainstem pathways.

### Parametric assessment of frequency-induced neuronal and hemodynamic responses in intermediolateral nucleus stimulation

To employ IML neural activity as a feedback variable in a CL algorithm, it must reliably reflect dynamic changes in blood pressure. To evaluate this suitability, we examined how neural activity and blood pressure respond to varying stimulation frequency (Fig. 2A&B). This analysis seeks to verify a causal relationship between stimulation input and physiological output and to assess whether IML neural signals are appropriate for feedback-based blood pressure regulation.

The experiments were conducted on seven normotensive Sprague-Dawley rats (from 7 rats, *n* ​= ​14). As stimulation frequency increased, the maximum reduction in blood pressure and the peak neuronal firing rate also increased (Fig. 2C&D). The magnitude of neural and blood pressure responses exhibited a proportional relationship with stimulation frequency, suggesting that higher frequencies can elicit stronger responses (Pearson correlation; *r* ​= ​0.71) (Fig. 2E). However, an increase in stimulation frequency did not consistently result in sustained reductions in blood pressure. Given the role of the IML in sympathetic regulation, the relationship between the mean firing rate at different stimulation frequencies and the blood pressure recovery time constant was examined. Higher stimulation frequencies were associated with a more rapid return of blood pressure to baseline (median, s: 4 ​Hz ​= ​62.50; 10 ​Hz ​= ​88.50; 20 ​Hz ​= ​62.00; 40 ​Hz ​= ​24.00), along with elevated mean neural activity (median, normalized value: 4 ​Hz ​= ​0.16; 10 ​Hz ​= ​0.32; 20 ​Hz ​= ​0.31; 40 ​Hz ​= ​0.50) (Fig. 2G&H). A moderate negative correlation was observed between stimulation frequency and the blood pressure recovery time constant (Pearson correlation; *r* ​= ​−0.42) (Fig. 2I). These findings indicate that neural activity within the right IML contributes to blood pressure recovery and compensatory mechanisms following stimulation. Furthermore, continuous stimulation at 40 ​Hz induced unintended elevations in blood pressure (mean ​± ​SD, mmHg: 4 ​Hz ​= ​0.47 ​± ​0.47; 40 ​Hz ​= ​5.45 ​± ​5.30) (Fig. 2J). Although higher stimulation frequencies produced greater reductions in blood pressure, the duration of the effect was shortened, and elevations above baseline were observed during the stimulation period. These findings emphasize that precise regulation of stimulation frequency is essential to maximize the duration of blood pressure reduction.

### Design and *in vivo* performance evaluation of the closed-loop stimulation strategy

A prior parametric study demonstrated that varying stimulation frequency influences blood pressure and neural responses, and based on this finding, a data-driven dynamic IO model was developed to predict IML neural responses from stimulation frequency, accurately capturing the neural activity. Using the separation principle, an LQG controller was subsequently designed, with weighting parameters optimized via simulation-based parameter adjustment (Supplementary Notes 3 & 4) (Fig. S8). To enhance safety, the weighting coefficient in the cost function was set high to slow convergence, and stimulation frequency was limited to below 30 ​Hz. The simulation confirmed that all stimulation remained within safe limits.

**Fig. 3:**
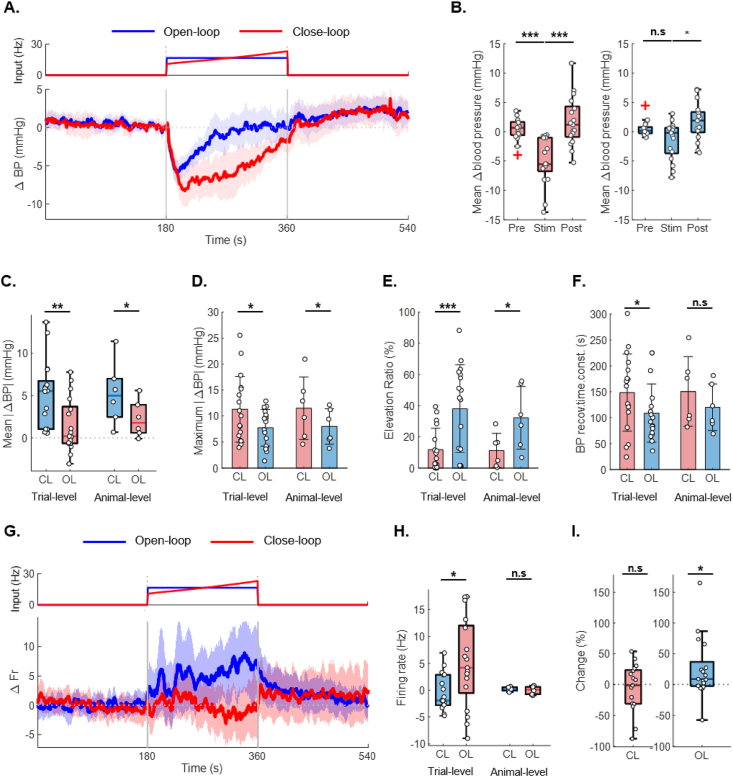
***In vivo* performance evaluation of the closed-loop stimulation algorithm. A.** Average blood pressure response with representative input. The blue line represents OL, and the red line represents CL (from 6 rats). **B.** Mean blood pressure reduction measured before, during, and after stimulation. **C.** Absolute reduction in mean blood pressure relative to baseline. D. Reduction in the minimum blood pressure during stimulation relative to baseline. **E.** Blood pressure elevation ratio, defined as the proportion of the stimulation period during which blood pressure exceeded baseline. **F.** Blood pressure recovery time constant. **G.** Average neural response with representative input. **H.** Mean change in firing rate during stimulation. **I.** Comparison of firing rates normalized over the 30 ​s before and after stimulation showed a significant increase in firing rate over time in the OL group. For panels A and G, data are shown over a 180-s window before and after stimulation. The mean values are plotted as lines, and the shaded areas represent the 95 ​% confidence intervals. For panels B, data are presented as boxplots (median, IQR, and 1.5 ​× ​IQR whiskers) with individual data points. Group differences were assessed using the Kruskal–Wallis test with Dunn–Šidák post hoc correction. Significant pairwise differences are denoted by asterisks (n.s., not significant; ∗*p* ​< ​0.05, ∗∗*p* ​< ​0.01, ∗∗∗*p* ​< ​0.001). or panel C, data are presented as boxplots (median, IQR, and 1.5 ​× ​IQR whiskers) with individual data points. For panels D, E, and F, data are presented as bar graphs (mean ​± ​SD) with individual data points. Group differences were assessed using the two-sided Wilcoxon signed-rank test (n.s., not significant; ∗*p* ​< ​0.05, ∗∗*p* ​< ​0.01, ∗∗∗*p* ​< ​0.001). For panels H and I, data are presented as boxplots (median, IQR, and 1.5 ​× ​IQR whiskers) with individual data points. Group differences were assessed using the two-sided Wilcoxon signed-rank test (n.s., not significant; ∗*p* ​< ​0.05, ∗∗*p* ​< ​0.01, ∗∗∗*p* ​< ​0.001). For panels C–H, both animal-level and trial-level statistical analyses were conducted to account for trial-by-trial fluctuations (from 6 rats, 2–3 trials per rat). Trial-level statistics are shown on the left (trials, *n* ​= ​17), and animal-level statistics are shown on the right (subjects, *n* ​= ​6). For panels I, trial-level statistics were analyzed taking trial-by-trial fluctuations into account. Abbreviations: OL, open-loop; CL, closed-loop.

To evaluate the performance of the algorithm, *in vivo* experiments were conducted. The experiments were performed on six normotensive rats, with one trial was terminated early due to excessive physical fatigue beyond the predefined threshold. (from 6 rats, *n* ​= ​17). To evaluate the blood pressure-lowering effects of each stimulation algorithm, the average change in blood pressure was calculated (Fig. 3A). Despite comparable stimulation intensities, OL stimulation did not produce a significant change in blood pressure, whereas CL stimulation resulted in a significant reduction (median, mmHg: CL-pre ​= ​0.65; CL-stim ​= ​−5.55; OL-pre ​= ​0.12; OL-stim ​= ​−0.19) (Fig. 3B&C). Furthermore, the maximum reduction in blood pressure was significantly greater in the CL group compared to the OL group (mean ​± ​SD, mmHg: CL ​= ​11.33 ​± ​6.34; OL ​= ​7.77 ​± ​6.35) (Fig. 3D). These results show that CL stimulation achieves a more effective reduction in blood pressure than OL stimulation. During the stimulation period, repeated elevations in blood pressure above baseline were observed, consistent with findings from the previous parameter study. To quantify this phenomenon, the “blood pressure elevation ratio” was defined as the proportion of time during which blood pressure increased during stimulation. This ratio was significantly higher under the OL condition, indicating that CL stimulation effectively suppressed undesirable side effects (mean ​± ​SD, %: CL ​= ​11.83 ​± ​13.61; OL ​= ​38.07 ​± ​28.23) (Fig. 3E). Analysis of the blood pressure recovery time constant showed that CL stimulation produced a significantly longer time constant compared to that of OL stimulation (mean ​± ​SD: CL ​= ​148.35 ​± ​74.58 ​s; OL ​= ​108.88 ​± ​56.25 ​s) (Fig. 3F). This finding indicates that blood pressure returned to baseline more slowly with CL stimulation, suggesting that CL stimulation effectively inhibited baroreflex-mediated recovery and thereby prolonged the antihypertensive effect. In contrast, OL stimulation, which employs fixed stimulation parameters, likely induces faster adaptation and a shorter effective duration. To further examine this mechanism, neural responses related to blood pressure regulation were analyzed. Neural activity was quantified as the change in firing rate from baseline, with CL stimulation producing a lower mean firing rate than that of OL stimulation (mean ​± ​SD, spikes/s: CL ​= ​−0.71 ​± ​2.84; OL ​= ​5.69 ​± ​7.72) (Fig. 3G & H). Under CL stimulation, the mean firing rate decreased below baseline, indicating suppression of IML neural activity. For detailed comparison of the two stimulation modes, the mean firing rate during the first 30 ​s after stimulation onset was defined as the “early” firing rate, and that during the last 30 ​s before stimulation termination was defined as the “late” firing rate. The ratio of the late to early firing rate was calculated for each subject, with a value of 1 indicating no change, to assess whether significant changes occurred (Median: CL ​= ​−1.408 ​%; OL ​= ​8.996 ​%) (Fig. 3I). Under OL stimulation, a significant increase in firing rate was observed, whereas no significant increase was detected with CL stimulation. These results show that the gradual increase in input frequency during CL stimulation suppressed the rise in IML firing rate—closely associated with compensatory mechanisms such as the baroreflex—thereby slowing blood pressure recovery.

At the animal level, statistical analyses of neural activity and blood pressure recovery tend to yield higher p-values, likely reflecting the influence of intrinsic neural dynamics, which may vary within the same subject even under identical stimulation conditions. Previous studies similarly reveal variability in evoked neural responses across trials, despite constant stimulation parameters and targeting, attributing this variability to fluctuations in the intrinsic state of the nervous system at the time of stimulation. To distinguish these intrinsic fluctuations from stimulation-induced effects, trial-level statistical analysis is essential. To more accurately capture the complexity of neural responses and their influence on blood pressure dynamics, trial-level analyses were conducted alongside animal-level analyses for all major parameters, including firing rate and blood pressure recovery.

### Relationship between neuronal activities in intermediolateral nucleus and blood pressure

Blood pressure regulation is governed by population-level dynamics that arise from interactions among neuronal ensembles, rather than the activity of individual neurons [58]. Previous studies show that multi-unit firing rates may be used to reliably estimate these dynamics without the need for spike sorting [59]. For implementing the CL stimulation algorithm, neural activity was recorded directly from the IML using a multichannel electrode, and the resulting signals were used as the feedback input. Since channel-level firing rates represent linear combinations of single-unit activities, they are suitable for estimating underlying population dynamics. The developed model effectively captured network-level dynamics. However, because multichannel electrodes record signals non-selectively, quantitative verification of their blood pressure–related information content is essential.

**Fig. 4:**
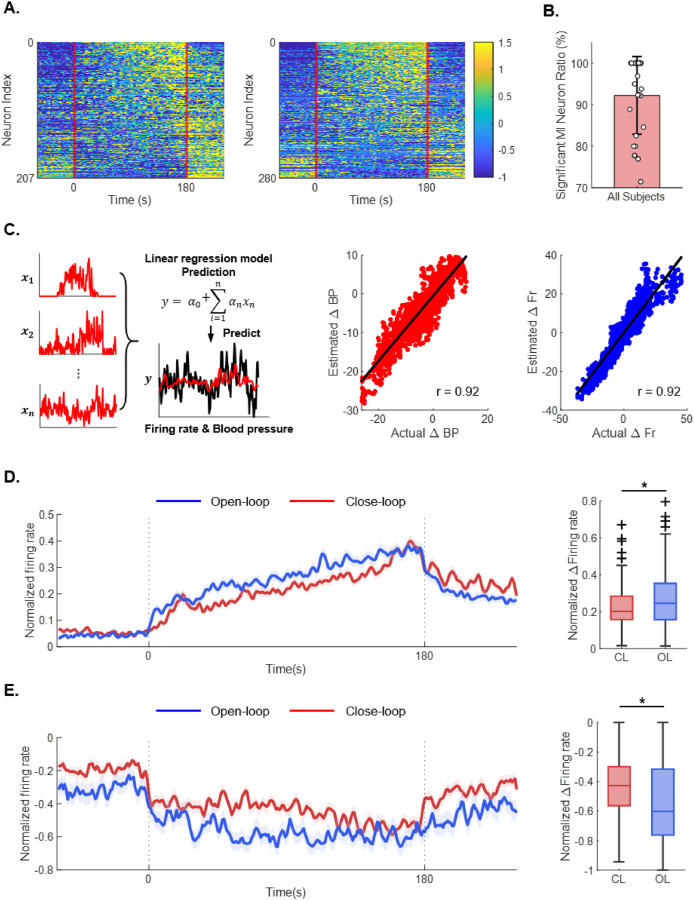
**Relationship between neuronal activity of intermediolateral nucleus and blood pressure. A.** Normalized neural activity heatmaps obtained through spike sorting for OL (left) and CL (right) stimulation conditions (from 5 rats, *n* ​= ​15). Firing rates were z-scored and displayed across the full recording duration. The stimulation period is indicated by two red vertical lines. **B.** Proportion of neurons encoding significant blood pressure–related information, calculated using mutual information. Data are presented as bar graphs (mean ​± ​SD) with individual data points. **C.** To determine whether neuronal activity contained sufficient information about blood pressure and firing rate, a linear regression model was applied to estimate these variables based on neuronal activity. The prediction accuracy was evaluated by calculating the Pearson correlation coefficient between the predicted and actual values across all data points. The middle panel shows the prediction of blood pressure from neuronal activity (red dots, 5040 data points), and the right panel shows the prediction of firing rate from neuronal activity (blue dots, 5040 data points). The black line represents the fitted linear regression line. **D.** Average response of individual excitatory neurons (left). The bold line represents the mean, and the shaded area indicates the 95 ​% confidence interval. Mean firing rate of individual excitatory neurons (right). Data are presented as boxplots (median, IQR, and 1.5 ​× ​IQR whiskers) with individual data points. Group differences were assessed using the two-sided Wilcoxon signed-rank test (n.s., not significant; ∗*p* ​< ​0.05, ∗∗*p* ​< ​0.01, ∗∗∗*p* ​< ​0.001). **E.** Average response of individual inhibitory neurons (left). The bold line represents the mean, and the shaded area indicates the 95 ​% confidence interval. Mean firing rate of individual inhibitory neurons (right). Data are presented as boxplots (median, IQR, and 1.5 ​× ​IQR whiskers) with individual data points. Group differences were assessed using the two-sided Wilcoxon signed-rank test (n.s., not significant; ∗*p* ​< ​0.05, ∗∗*p* ​< ​0.01, ∗∗∗*p* ​< ​0.001). Abbreviations: OL, open-loop; CL, closed-loop; IML, intermediolateral nucleus.

To determine whether single IML neurons encode information about blood pressure recovery, spike sorting was performed, and single-unit activity was analyzed (Fig. 4A). Spike sorting was applied across all channels, with one subject excluded owing to persistent sorting errors. Mutual-information analysis was then conducted on the remaining datasets (*n* ​= ​10) to assess the association between mean IML activity and blood pressure recovery. Overall, 93.2 ​% of neurons exhibited statistically significant mutual information with blood pressure (mean ​± ​SD: 92.72 ​± ​8.99 ​%), indicating that these neurons convey relevant information about recovery (Fig. 4B). For compatibility with implantable devices, only 4 of the 16 available channels were used to construct the feedback feature. Because a firing rate of a channel represents the activity of nearby neurons, an additional analysis was performed to test whether channel-level firing could be employed to reconstruct single-unit activity encoding blood pressure information. The predicted single-unit firing rates exhibited a strong positive correlation with the measured values, and blood pressure predictions were similarly well correlated with the actual measurements (predicted firing rate: *r* ​= ​0.925; predicted blood pressure: *r* ​= ​0.918; Pearson’s r) (Fig. 4C). These results show that IML signals recorded with a multichannel electrode provide sufficient information to approximate single-unit activity and that individual neurons encode meaningful blood-pressure–related information.

Considering the anatomical location and functional role of the IML, along with the previously observed negative correlation between mean IML firing rate and the blood-pressure recovery time constant, IML activity may reflect activation of compensatory mechanisms, such as the baroreflex, following a blood-pressure drop. To investigate this hypothesis, neurons were classified as excitatory or inhibitory based on their responses to the 180-s stimulation, and the two stimulation modes were compared (Median: Excitatory: CL ​= ​0.202, OL ​= ​0.245; Inhibitory: CL ​= ​−0.424, OL ​= ​0.603) (Fig. 4D and E). Compared with OL stimulation, CL stimulation reduced activity in excitatory and inhibitory neurons. These results show that CL does not selectively enhance a specific neuronal subset but instead broadly suppresses overall neuronal activity. As most neurons exhibited significant mutual information with blood pressure, the collective ensemble dynamics are likely integral to blood-pressure regulation. The relatively lower excitatory and inhibitory activity observed during CL suggests that this approach may more effectively suppress compensatory recovery mechanisms—such as the baroreflex—thereby promoting a more sustained reduction in blood pressure.

### Performance assessment of closed-loop stimulation strategy in Angiotensin-II induced hypertension

**Fig. 5:**
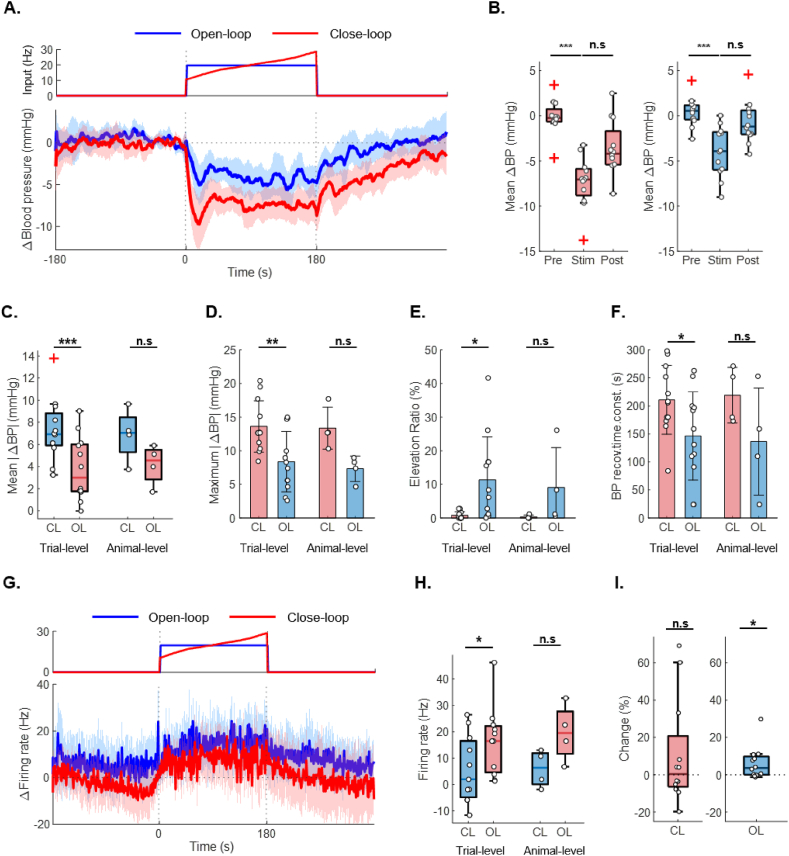
**Performance assessment of closed-loop stimulation strategy in Angiotensin-II induced hypertension. A.** Averaged blood pressure responses for OL (blue) and CL (red) stimulation. **B.** Mean blood pressure reduction measured before, during, and after stimulation (*n* ​= ​4). **C.** Absolute value of the mean blood pressure reduction. **D.** Maximum blood pressure change recorded during stimulation. **E.** Blood pressure elevation ratio, defined as the proportion of the stimulation period during which blood pressure exceeded baseline. **F.** Blood pressure recovery time constant. **G.** Average neural response for OL (blue) and CL (red) stimulation. Due to the characteristics of the TDT system, the signal during the first 3 ​s of recording was unreliable and therefore excluded from the analysis. **H.** Mean change in firing rate during stimulation. **I.** Comparison of firing rates normalized over the 30 ​s before and after stimulation showed a significant increase in firing rate over time in the OL group. For panels A and G, data are shown over a 180-s window before and after stimulation. The mean values are plotted as lines, and the shaded areas represent the 95 ​% confidence intervals. For panels B, data are presented as boxplots (median, IQR, and 1.5 ​× ​IQR whiskers) with individual data points. Outliers are denoted by red “+”. Group differences were assessed using the Kruskal–Wallis test with Dunn–Šidák post hoc correction. Significant pairwise differences are denoted by asterisks (n.s., not significant; ∗*p* ​< ​0.05, ∗∗*p* ​< ​0.01, ∗∗∗*p* ​< ​0.001). For panel C, data are presented as boxplots (median, IQR, and 1.5 ​× ​IQR whiskers) with individual data points. For panels D, E and F, data are presented as bar graphs (mean ​± ​SD) with individual data points. Group differences were assessed using the two-sided Wilcoxon signed-rank test (n.s., not significant; ∗*p* ​< ​0.05, ∗∗*p* ​< ​0.01, ∗∗∗*p* ​< ​0.001). For panels H and I, data are presented as boxplots (median, IQR, and 1.5 ​× ​IQR whiskers) with individual data points. Group differences were assessed using the two-sided Wilcoxon signed-rank test (n.s., not significant; ∗*p* ​< ​0.05, ∗∗*p* ​< ​0.01, ∗∗∗*p* ​< ​0.001). For panels C–H, both animal-level and trial-level statistical analyses were conducted to account for trial-by-trial fluctuations (from 4 rats, 3 trials per rat). Trial-level statistics are shown on the left (trials, *n* ​= ​12; for G, *n* ​= ​11), and animal-level statistics are shown on the right (subjects, *n* ​= ​4). Abbreviations: OL, open-loop; CL, closed-loop. BP, blood pressure.

The efficacy of the proposed CL stimulation strategy was further evaluated in the Angiotensin–II–induced hypertensive rat model. One rat died due to anesthesia-related complications during the procedure, and the experiment was conducted on the remaining four out of five successfully modeled rats. (from 4 rats, *n* ​= ​12) As in the normotensive experiments, the average stimulation frequency was matched between the OL and CL protocols to minimize the influence of stimulation intensity. Unlike in the normotensive group, CL and OL stimulation produced significant reductions in blood pressure during the stimulation period in the hypertensive group (median, mmHg: CL-pre ​= ​−0.24, CL-stim ​= ​−7.05; OL-pre ​= ​0.53, OL-stim ​= ​−3.91) (Fig. 5A and B). The average reduction in blood pressure was approximately twice as high in the CL group (mean ​± ​SD, mmHg: CL: 7.26 ​± ​2.83; OL ​= ​3.81 ​± ​2.88) (Fig. 5C), and the maximum reduction in blood pressure was also significantly elevated with CL stimulation (Fig. 5D). The proportion of blood pressure elevation during stimulation was markedly lower in stimulation protocols compared to that of the normotensive group, with the CL group exhibiting a value significantly closer to zero (mean ​± ​SD, %: CL ​= ​0.74 ​± ​1.09; OL ​= ​11.34 ​± ​12.75) (Fig. 5E). The blood pressure recovery time constant was approximately 44 ​% higher in the CL group (mean ​± ​SD, s: CL ​= ​219.33 ​± ​61.37; OL ​= ​147.75 ​± ​78.79) (Fig. 5F).

With respect to neural responses, the analysis was performed after excluding one noisy trial, resulting in a total of 11 trials from 4 animals (*n* ​= ​11). The average neural activity was significantly lower in the CL group compared to the OL group (mean ​± ​SD, spike/s: CL ​= ​5.66 ​± ​12.81; OL: 16.39 ​± ​13.14) (Fig. 5G and H). This result is consistent with the findings from the normotensive experiments and suggests that CL stimulation may suppress the activity of neurons involved in recovery. Furthermore, as in the normotensive experiments, changes in neural activity during stimulation were examined (Median: CL ​= ​0.407 ​%, OL ​= ​3.546 ​%) (Fig. 5I). In OL stimulation, neural activity exhibited a small but significant increase, whereas CL showed no significant change. Taken together with the earlier observation that firing rates were higher under OL than CL, this pattern may reflect the time-dependent activation of neurons previously identified, through spike sorting analysis, as being associated with blood pressure recovery. Due to the small sample size, statistical significance could not be claimed in the animal-level analysis; however, the observed trend was consistent with that in the normotensive experiments, suggesting that statistical significance could likely be achieved with a sufficient number of experiments.

Although the results cannot be fully generalized owing to limitations such as small sample size and variations in stimulation amplitude, a comparison of blood pressure responses between the hypertensive and normotensive groups revealed a greater overall reduction in the hypertensive model (mean ​± ​SD, mmHg: Normal: 5.15 ​± ​3.95; HTN: 7.26 ​± ​2.83), along with a more prolonged response duration (mean ​± ​SD, s: Normal: 152.67 ​± ​76.46; HTN: 210.92 ​± ​61.37). Previous studies have reported that higher baseline blood pressure is often associated with greater blood pressure changes [34], suggesting that the elevated baseline in the hypertensive group may have contributed to these observations.

## Discussion

The human autonomic nervous system regulates blood pressure through the opposing actions of the sympathetic and parasympathetic branches [60]. In hypertension, this balance becomes disrupted, with excessive sympathetic activity emerging as a key pathological factor. Therefore, neuromodulation strategies that aim to normalize sympathetic overactivity are gaining attention as potential alternatives to conventional drug therapies, which are often limited by side effects and suboptimal efficacy [19]. Although most previous neuromodulation studies for blood pressure control have targeted supraspinal structures such as the baroreflex pathway, these approaches have shown limited therapeutic benefit and are associated with adverse effects and surgical complications. The spinal cord functions as the primary conduit for descending sympathetic inputs from the brain and regulates sympathetic output to peripheral organs, making it a central node in blood pressure regulation. Recent studies reporting blood pressure reduction following spinal cord stimulation support targeting the spinal cord for neuromodulation [[32], [33], [34]]. To improve therapeutic efficiency and enable more precise regulation, a CL stimulation strategy was designed using neural activity from the IML—a region strongly associated with sympathetic output—as the feedback variable.

The CL stimulation strategy was developed based on the hypothesis that neural activity within the IML is closely associated with blood pressure regulation and can serve as an effective feedback variable for blood pressure control. To evaluate this hypothesis, a mutual information analysis was conducted to assess the relationship between the activity of individual IML neurons and blood pressure signals. The analysis revealed that approximately 93.2 ​% of the recorded neurons exhibited statistically significant correlations with blood pressure fluctuations, indicating that the IML encodes substantial information relevant to blood pressure regulation and is a viable candidate for feedback control in neuromodulation systems. However, owing to limitations in processor performance, it was not feasible to reliably extract individual neuronal activity in real-time employing spike sorting. As an alternative, this study utilized a neural signal composed of the summed firing rates from four selected channels within the 16-channel array. The predicted values from the linear regression model trained on individual neuronal activity exhibited a very strong correlation with the actual summed firing rates from the four selected channels, suggesting that this aggregated signal serves as a stable and interpretable input variable suitable for the CL algorithm. If real-time processing of single-neuron activity becomes achievable, it may enable the development of more precise and sophisticated stimulation algorithms.

**Fig. 6:**
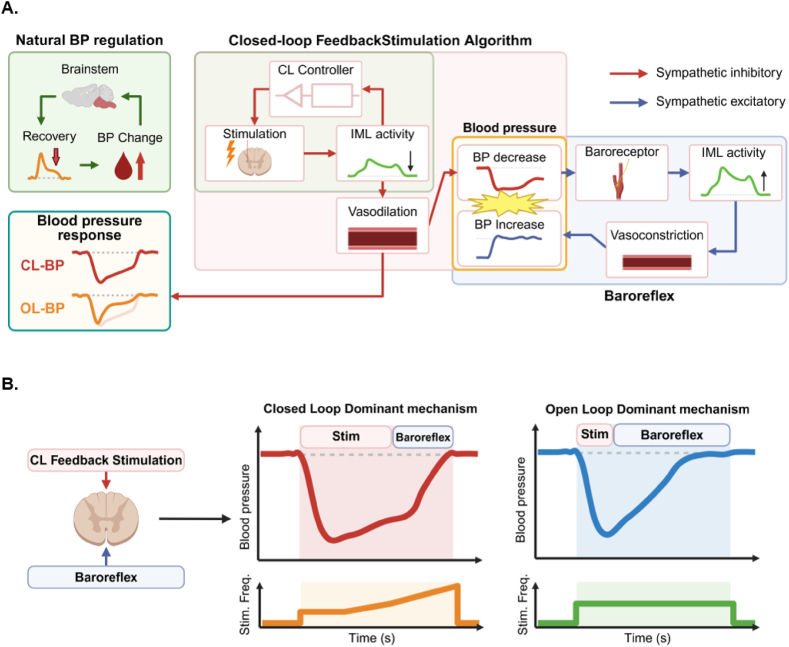
**Mechanistic Interpretation of suggested neuroprosthetic closed-loop stimulation strategy. A.** The Composition of spinal cord simulation and baroreflex. CL feedback simulation algorithm is designed to mimic natural blood pressure control mechanisms. **B.** Differences in the dominant mechanism between OL and CL stimulation. Abbreviations: OL, open-loop; CL, closed-loop.

To enhance the magnitude and duration of blood pressure reduction induced by stimulation, a CL stimulation strategy was developed to automatically adjust stimulation intensity based on neural activity in the IML. The system incorporates a custom real-time artifact removal algorithm, enabling accurate estimation of neuronal firing rates without distortion from electrical stimulation. The control algorithm was implemented utilizing an LQG controller that was designed based on the model and integrates a Kalman filter to improve robustness against noise, which is an inherent challenge in physiological signal acquisition. While model-based control strategies have been extensively explored in neuromodulation research, most prior studies have been confined to simulations, with limited implementation in biological systems. In contrast, this study demonstrates a model-based control strategy that was validated with experimental data in a physiological setting, including a hypertension model. In normotensive and hypertensive models, CL stimulation produced a greater and more sustained reduction in blood pressure than that of OL stimulation. This superior effect is attributable to the use of an LQG controller within the CL system, which continuously adjusts stimulation input based on real-time IML neural activity. Under CL stimulation, the increase in mean firing rate was comparatively smaller and did not exhibit a progressive rise over the course of stimulation. Furthermore, spike sorting was employed to classify neurons into excitatory and inhibitory groups, and analysis of stimulation-induced activity revealed consistently lower activity in both populations under CL stimulation. These findings reveal that continuous frequency modulation based on neural activity in the CL approach enhances the persistence of stimulation effects and more effectively suppresses compensatory mechanisms for blood pressure regulation, such as the baroreflex. In contrast, OL stimulation, which relies on fixed parameters, is more susceptible to neural adaptation, thereby reducing the durability of its effects (Fig. 6). In this study, the average stimulation intensity for OL was matched to that of CL to ensure a fair comparison; however, in clinical settings, OL parameters are typically adjusted manually by clinicians, which may further constrain their effectiveness. These findings support the therapeutic durability, efficacy, and clinical applicability of the CL stimulation strategy.

A synthesis of existing literature and the current findings reveals that SCS may function not merely as a means of regulating blood pressure but also as a therapeutic strategy for restoring autonomic balance. Previous studies have reported that eSCS reduces blood pressure in patients with chronic pain or hypertension by activating inhibitory interneurons, thereby suppressing sympathetic activity and inducing vasodilation [[32], [33], [34]]. Consistent with previous findings, this study demonstrated that SCS induced vasodilation, leading to a reduction in blood pressure in normotensive and hypertensive models. These results support the idea that SCS contributes to stabilizing function by suppressing excessive sympathetic output within normal spinal circuits. In contrast, patients with spinal cord injury often experience orthostatic hypotension owing to the disrupted supraspinal vasomotor control, which inhibits signals from reaching the spinal sympathetic circuits, specifically the SPNs [61]. Under these pathological conditions, eSCS enhances sympathetic output and restores blood pressure [23,62], thereby alleviating orthostatic hypotension. This excitatory response can be attributed to the maladaptive reorganization of spinal circuits over time, characterized by the abnormal strengthening of excitatory connections between interneurons and SPNs [63]. These findings reveal that the efficacy of SCS may vary depending on the structural and functional state of the spinal circuitry. Therefore, SCS has the potential to serve as a therapeutic approach for conditions associated with autonomic dysregulation, including hypertension.

Implantable neurostimulators targeting the spinal cord are now widely applied in clinical practice, particularly for restoring motor function following spinal cord injury [64,65] and managing chronic pain [66,67], demonstrating the clinical safety and efficacy of SCS technologies. These precedents suggest that the CL SCS approach proposed in this study may also be clinically applicable for the treatment of cardiovascular diseases, provided that appropriate technological refinements and noninvasive signal acquisition methods are established.

Despite its contributions, this study has some limitations. First, owing to the challenges in establishing the hypertensive model, the sample size was relatively small, and statistical analyses at the animal level did not yield significant p-values. However, trial-level analyses that accounted for trial-to-trial fluctuations produced significant results, suggesting that a larger sample size may enable statistically significant outcomes even at the animal level. Second, the stimulation duration was restricted to 3 ​min to emphasize differences between CL and OL during acute stimulation. A major limitation of this study is the lack of evaluation of blood pressure changes under longer stimulation durations. For more prolonged stimulation, it may be necessary to adjust the input weights of the controller to more precisely regulate the rate of change in input. Given that many clinical studies apply stimulation continuously on a daily basis [68,69], future investigations should assess how extending the stimulation time per treatment session influences outcomes and examine the effects of multi-week stimulation on the cardiovascular system. Finally, in this study, invasive electrodes were used to precisely record neural activity in the IML, a procedure associated with considerable clinical risk. The IML plays a critical physiological role in blood pressure regulation; however, its location within the lateral horn of the spinal cord presents significant anatomical challenges for noninvasive signal access [70]. Consequently, the development and validation of technologies that can reliably and stably detect blood pressure–related neural signals through noninvasive means remain a key challenge for the clinical translation of the CL stimulation approach. Recent advances in techniques for noninvasively recording neural signals from the spinal surface have been progressing rapidly, and the potential for detecting blood pressure–related signals through such methods is becoming increasingly promising [[71], [72], [73]]. These technological developments offer a feasible pathway for translating the CL SCS-based neuromodulation strategy proposed in this study into clinical applications.

## Author contributions

M.G.C. M.H.C, S.M.P contributed to the study conceptualization and design. M.G.C, M.H.C, M.T.K, S.U.H, C.H.O performed all the experiments and collected the data. J.S.M, J.H.K provided technical expertise. M.G.C, M.H.C, M.T.K analyzed data. All authors discussed the results and commented on the manuscript.

## Data and materials availability

All data needed to evaluate the conclusions in the paper are present in the paper and/or the Supplementary Materials.

## Source of funding

This research was supported by the Bio&Medical Technology Development Program of the National Research Foundation (NRF) funded by the Korean government (MSIT) (No. RS-2024-00361688), Pioneer Research Center Program through the National Research Foundation of Korea funded by the Ministry of Science, ICT & Future Planning (2022M3C1A3081294), the University Technology Commercialization Promotion Program through the Commercializations Promotion Agency for R&D Outcomes (COMPA) funded by the National Research Foundation of Korea (NRF) (RS-2024-00426901), National R&D Program through the National Research Foundation of Korea (NRF) funded by Ministry of Science and ICT (2021M3H4A1A03049084), the National Research Foundation of Korea (NRF) grant funded by the Korea government (MSIT) (RS-2025-00517742).

## Declaration of Competing Interest

The authors declare that they have no known competing financial interests or personal relationships that could have appeared to influence the work reported in this paper.

## References

[1] (2021). Worldwide trends in hypertension prevalence and progress in treatment and control from 1990 to 2019: a pooled analysis of 1201 population-representative studies with 104 million participants. Lancet.

[2] Kario K., Okura A., Hoshide S., Mogi M. (2024). The WHO global report 2023 on hypertension warning the emerging hypertension burden in globe and its treatment strategy. Hypertens Res.

[3] Esler M., Straznicky N., Eikelis N., Masuo K., Lambert G., Lambert E. (2006). Mechanisms of sympathetic activation in obesity-related hypertension. Hypertension.

[4] Grassi G., Mark A., Esler M. (2015). The sympathetic nervous system alterations in human hypertension. Circ Res.

[5] DeLalio L.J., Sved A.F., Stocker S.D. (2020). Sympathetic nervous system contributions to hypertension: updates and therapeutic relevance. Can J Cardiol.

[6] Zeltser H.K.R. (2024 2024).

[7] Unger T., Borghi C., Charchar F., Khan N., Poulter N., Prabhakaran D. (2020).

[8] Hamrahian S.M. (2020). Medication non-adherence: a major cause of resistant hypertension. Curr Cardiol Rep.

[9] Tedla Y.G., Bautista L.E. (2016). Drug side effect symptoms and adherence to antihypertensive medication. Am J Hypertens.

[10] Gebreyohannes E.A., Bhagavathula A.S., Abebe T.B., Tefera Y.G., Abegaz T.M. (2019). Adverse effects and non-adherence to antihypertensive medications in university of gondar comprehensive specialized hospital. Clin Hypertens.

[11] Burnier M., Egan B.M. (2019). Adherence in hypertension. Circ Res.

[12] O’Callaghan E.L., McBryde F.D., Burchell A.E., Ratcliffe L.E., Nicolae L., Gillbe I. (2014). Deep brain stimulation for the treatment of resistant hypertension. Curr Hypertens Rep.

[13] Mahfoud F., Schlaich M.P., Lobo M.D. (2021). Device therapy of hypertension. Circ Res.

[14] Krum H., Sobotka P., Mahfoud F., Böhm M., Esler M., Schlaich M. (2011). Device-based antihypertensive therapy. Circulation.

[15] Bisognano J.D., Bakris G., Nadim M.K., Sanchez L., Kroon A.A., Schafer J. (2011). Baroreflex activation therapy lowers blood pressure in patients with resistant hypertension: results from the double-blind, randomized, placebo-controlled rheos pivotal trial. J Am Coll Cardiol.

[16] de Leeuw P.W., Bisognano J.D., Bakris G.L., Nadim M.K., Haller H., Kroon A.A. (2017). Sustained reduction of blood pressure with baroreceptor activation therapy: results of the 6-Year open Follow-Up. Hypertension.

[17] O’Callaghan E.L., Hart E.C., Sims-Williams H., Javed S., Burchell A.E., Papouchado M. (2017). Chronic deep brain stimulation decreases blood pressure and sympathetic nerve activity in a Drug- and device-resistant hypertensive patient. Hypertension.

[18] Green A.L., Wang S., Owen S.L.F., Xie K., Liu X., Paterson D.J. (2005). Deep brain stimulation can regulate arterial blood pressure in awake humans. Neuroreport.

[19] Mun J., Lee J., Park S.-M. (2024). Real-time closed-loop brainstem stimulation modality for enhancing temporal blood pressure reduction. Brain Stimul.

[20] Lozano A.M., Lipsman N., Bergman H., Brown P., Chabardes S., Chang J.W. (2019). Deep brain stimulation: current challenges and future directions. Nat Rev Neurol.

[21] Scheffers I.J., Kroon A.A., de Leeuw P.W. (2010). Carotid baroreflex activation: past, present, and future. Curr Hypertens Rep.

[22] Minic Z., O’Leary D.S., Reynolds C.A. (2022). Spinal reflex control of arterial blood pressure: the role of TRP channels and their endogenous eicosanoid modulators. Front Physiol.

[23] Squair J.W., Gautier M., Mahe L., Soriano J.E., Rowald A., Bichat A. (2021). Neuroprosthetic baroreflex controls haemodynamics after spinal cord injury. Nature.

[24] Strack A.M., Sawyer W.B., Marubio L.M., Loewy A.D. (1988). Spinal origin of sympathetic preganglionic neurons in the rat. Brain Res.

[25] Deuchars S.A., Lall V.K. (2015). Sympathetic preganglionic neurons: properties and inputs. Compr Physiol.

[26] Morrison S.F. (2001). Differential control of sympathetic outflow. Am J Physiol Regul Integr Comp Physiol.

[27] Dampney R.A.L. (2017). Resetting of the Baroreflex control of sympathetic vasomotor activity during natural behaviors: description and conceptual model of central mechanisms. Front Neurosci.

[28] Biaggioni I., Shibao C.A., Diedrich A., Muldowney J.A.S., Laffer C.L., Jordan J. (2019). Blood pressure management in afferent baroreflex failure: JACC review topic of the week. J Am Coll Cardiol.

[29] Oshima N., Kumagai H., Onimaru H., Kawai A., Pilowsky P.M., Iigaya K. (2008). Monosynaptic excitatory connection from the rostral ventrolateral medulla to sympathetic preganglionic neurons revealed by simultaneous recordings. Hypertens Res.

[30] Zanutto B.S., Valentinuzzi M.E., Segura E.T. (2010). Neural set point for the control of arterial pressure: role of the nucleus tractus solitarius. Biomed Eng Online.

[31] Linderoth B., Foreman R.D. (1999). Physiology of spinal cord stimulation: review and update. Neuromodulation: Technol Neural Interf.

[32] Holwerda S.W., Holland M.T., Green A.L., Pearson A.C.S., Pierce G.L. (2021). Dissociation between reduced pain and arterial blood pressure following epidural spinal cord stimulation in patients with chronic pain: a retrospective study. Clin Auton Res.

[33] Holwerda S.W., Holland M.T., Green A.L., Collins M.T., Pearson A.C., Pierce G.L. (2019). Epidural spinal cord stimulation for neuropathic pain reduces blood pressure in patients with hypertension independent of pain relief: a retrospective study. FASEB J.

[34] Memar K., Varghese S.N., Morrison A.G., Clonch D.A., Lam C.M., Holwerda S.W. (2023). Low- and high-frequency spinal cord stimulation and arterial blood pressure in patients with chronic pain and hypertension: a retrospective study. Clin Auton Res.

[35] Plachta D.T., Gierthmuehlen M., Cota O., Espinosa N., Boeser F., Herrera T.C. (2014). Blood pressure control with selective vagal nerve stimulation and minimal side effects. J Neural Eng.

[36] Botzer A., Finkelstein Y., Unger R. (2021). Blood pressure regulation evolved from basic homeostatic components. Biomedicines.

[37] Domingos-Souza G., Santos-Almeida F.M., Meschiari C.A., Ferreira N.S., Pereira C.A., Pestana-Oliveira N. (2021). The ability of baroreflex activation to improve blood pressure and resistance vessel function in spontaneously hypertensive rats is dependent on stimulation parameters. Hypertens Res.

[38] O’Callaghan E.L., Hart E.C., Sims-Williams H., Javed S., Burchell A.E., Papouchado M. (2017). Chronic deep brain stimulation decreases blood pressure and sympathetic nerve activity in a Drug- and device-resistant hypertensive patient. Hypertension.

[39] Grassi G., Seravalle G., Stella M.L., Turri C., Zanchetti A., Mancia G. (2000). Sympathoexcitatory responses to the acute blood pressure fall induced by central or peripheral antihypertensive drugs. Am J Hypertens.

[40] Duarte R.V., Bresnahan R., Copley S., Eldabe S., Thomson S., North R.B. (2024). Reporting guidelines for randomised controlled trial reports of implantable neurostimulation devices: the CONSORT-iNeurostim extension. eClinicalMedicine.

[41] Nentwich M., Leszczynski M., Schroeder C.E., Bickel S., Parra L.C. (2025). Intrinsic dynamic shapes responses to external stimulation in the human brain. eLife.

[42] Yang Y, Qiao S, Sani OG, Sedillo JI, Ferrentino B, Pesaran B, et al. Modelling and prediction of the dynamic responses of large-scale brain networks during direct electrical stimulation. Nat Biomed Eng. 2021, 0201 ed2021. p. 324-345. 10.1038/s41551-020-00666-w 33526909

[43] Scangos K.W., Makhoul G.S., Sugrue L.P., Chang E.F., Krystal A.D. (2021). State-dependent responses to intracranial brain stimulation in a patient with depression. Nat Med.

[44] Pasley B.N., Allen E.A., Freeman R.D. (2009). State-dependent variability of neuronal responses to transcranial magnetic stimulation of the visual cortex. Neuron.

[45] Idesis S., Allegra M., Vohryzek J., Sanz Perl Y., Faskowitz J., Sporns O. (2023). A low dimensional embedding of brain dynamics enhances diagnostic accuracy and behavioral prediction in stroke. Sci Rep.

[46] Edhi M.M., Heijmans L., Vanent K.N., Bloye K., Baanante A., Jeong K.-S. (2020). Time-dynamic pulse modulation of spinal cord stimulation reduces mechanical hypersensitivity and spontaneous pain in rats. Sci Rep.

[47] Avendaño-Coy J., Bravo-Esteban E., Ferri-Morales A., Martínez-de la Cruz R., Gómez-Soriano J. (2019). Does frequency modulation of transcutaneous electrical nerve stimulation affect habituation and mechanical hypoalgesia? A randomized, double-blind, Sham-Controlled crossover trial. Phys Ther.

[48] Wilmerding L.K., Yazdanbakhsh A., Hasselmo M.E. (2022). Impact of optogenetic pulse design on CA3 learning and replay: a neural model. Cell Rep Method.

[49] Nair A.B., Jacob S. (2016). A simple practice guide for dose conversion between animals and human. J Basic Clin Pharm.

[50] Crowley S.D., Gurley S.B., Herrera M.J., Ruiz P., Griffiths R., Kumar A.P. (2006). Angiotensin II causes hypertension and cardiac hypertrophy through its receptors in the kidney. Proc Natl Acad Sci USA.

[51] Zhan Y., Brown C., Maynard E., Anshelevich A., Ni W., Ho I.C. (2005). Ets-1 is a critical regulator of Ang II-mediated vascular inflammation and remodeling. J Clin Investig.

[52] Kuroki M.T., Fink G.D., Osborn J.W. (2014). Comparison of arterial pressure and plasma ANG II responses to three methods of subcutaneous ANG II administration. Am J Physiol Heart Circ Physiol.

[53] Granata A.R., Kitai S.T. (1992). Intracellular analysis *in vivo* of different barosensitive bulbospinal neurons in the rat rostral ventrolateral medulla. J Neurosci.

[54] Morrison S.F., Callaway J., Milner T.A., Reis D.J. (1989). Glutamate in the spinal sympathetic intermediolateral nucleus: localization by light and electron microscopy. Brain Res.

[55] Tanaka S., Barron K.W., Chandler M.J., Linderoth B., Foreman R.D. (2001). Low intensity spinal cord stimulation may induce cutaneous vasodilation via CGRP release. Brain Res.

[56] Cooper C.M., Farrand A.Q., Andresen M.C., Beaumont E. (2021). Vagus nerve stimulation activates nucleus of solitary tract neurons via supramedullary pathways. J Physiol.

[57] Waxenbaum J.A., Reddy V., Varacallo M.A. (2025).

[58] Guyenet P.G., Stornetta R.L., Souza G., Abbott S.B.G., Brooks V.L. (2020). Neuronal networks in hypertension: recent advances. Hypertension.

[59] Trautmann E.M., Stavisky S.D., Lahiri S., Ames K.C., Kaufman M.T., O’Shea D.J. (2019). Accurate estimation of neural population dynamics without spike sorting. Neuron.

[60] Joyner M.J., Charkoudian N., Wallin B.G. (2010). Sympathetic nervous system and blood pressure in humans: individualized patterns of regulation and their implications. Hypertension.

[61] Claydon V.E., Steeves J.D., Krassioukov A. (2006). Orthostatic hypotension following spinal cord injury: understanding clinical pathophysiology. Spinal Cord.

[62] Beliaeva N.N., Moshonkina T.R., Mamontov O.V., Zharova E.N., Condori Leandro H.I., Gasimova N.Z. (2022). Transcutaneous spinal cord stimulation attenuates blood pressure drops in orthostasis. Life.

[63] Schramm L.P., Weaver L.C., Polosa C. (2006).

[64] Davis K.C., Wyse-Sookoo K.R., Raza F., Meschede-Krasa B., Prins N.W., Fisher L. (2025). 5-year follow-up of a fully implanted brain-computer interface in a spinal cord injury patient. J Neural Eng.

[65] Darrow D., Balser D., Netoff T.I., Krassioukov A., Phillips A., Parr A. (2019). Epidural spinal cord stimulation facilitates immediate restoration of dormant motor and autonomic supraspinal pathways after chronic neurologically complete spinal cord injury. J Neurotrauma.

[66] Brooker C., Russo M., Cousins M.J., Taylor N., Holford L., Martin R. (2021). ECAP-Controlled closed-loop spinal cord stimulation efficacy and opioid reduction over 24-Months: final results of the prospective, multicenter, open-label avalon study. Pain Pract.

[67] North R.B., Kidd D.H., Zahurak M., James C.S., Long D.M. (1993). Spinal cord stimulation for chronic, intractable pain: experience over two decades. Neurosurgery.

[68] González-Zamorano Y., Sánchez-Cuesta F., Moreno M., Arroyo Ferrer A., Fernández-Carnero J., Ray Chaudhuri K. (2024). TDCS for Parkinsońs disease-related pain: a randomized trial. Clin Neurophysiol.

[69] Pavlova E.L., Menshikova A.A., Semenov R.V., Bocharnikova E.N., Gotovtseva G.N., Druzhkova T.A. (2018). Transcranial direct current stimulation of 20- and 30-minutes combined with sertraline for the treatment of depression. Prog Neuropsychopharmacol Biol Psychiat.

[70] Watson C., Paxinos G., Kayalioglu G., Heise C., Watson C., Paxinos G., Kayalioglu G. (2009). The spinal cord.

[71] Woodington B.J., Lei J., Carnicer-Lombarte A., Güemes-González A., Naegele T.E., Hilton S. (2024). Flexible circumferential bioelectronics to enable 360-degree recording and stimulation of the spinal cord. Sci Adv.

[72] Burke J.F., Kunwar N., Yaroshinsky M.S., Louie K.H., Shirvalkar P., Su P. (2021). Epidural spinal electrogram provides direct spinal recordings in awake human participants. Front Hum Neurosci.

[73] Chander B.S., Deliano M., Azañón E., Büntjen L., Stenner M.-P. (2022). Non-invasive recording of high-frequency signals from the human spinal cord. Neuroimage.

