# Supplementary material for: Neuroprosthetic closed-loop strategy for sustained blood pressure reduction via simultaneous stimulation and recording from the spinal cord
Source: Neurotherapeutics. 2025 Sep 30;22(6):e00758. doi: 10.1016/j.neurot.2025.e00758 (PMC12664564; doi:10.1016/j.neurot.2025.e00758)
Supplement: Multimedia component 1 [file mmc1.pdf]

Supplementary Note 1. Artifact Removal Methodology

Stimulation-induced artifacts are a major obstacle to accurate neural signal analysis, making artifact removal essential for reliable estimation of neural activity [1]. To minimize signal distortion, the original signal was duplicated and artifacts were removed using an interpolation-based method [2] (Figure S3A). Considering the duration of the artifacts, a removal window of 192 samples (approximately 0.08 ms) was applied.

To quantitatively assess the degree of signal distortion introduced by the artifact removal process, data from the resting period (5 minutes) before stimulation in all experiments were used. Artificial pulses at 30 Hz and 40 Hz were inserted into this segment (n = 15), and the same artifact removal algorithm was applied to each pulse. Firing rates were then calculated, and the changes in firing rate before and after artifact removal were compared to evaluate signal distortion.

The results showed no significant changes in firing rate under both 30 Hz and 40 Hz conditions, indicating that signal distortion due to artifact removal was minimal. Based on this, the upper limit of stimulation frequency for actual experiments was set to below 30 Hz for the normotensive group and below 40 Hz for the hypertensive model (Figure S3B&C).

Supplementary Note 2. Biopsy result of Angiotensin-II induced hypertension model

To confirm successful induction of hypertension, blood pressure was continuously monitored using the tail-cuff method following osmotic pump implantation. To minimize stress- and movement-induced fluctuations, measurements were performed under inhalation anesthesia. Among the six rats implanted with the pump, one was excluded from the analysis due to early

pump migration, which prevented the expected initial elevation in blood pressure (Figure S5A). Following implantation, blood pressure increased progressively, and by the third week, a statistically significant elevation was observed compared to pre-implantation levels (Figure S5B).

In addition to blood pressure measurements, histological analyses were conducted to verify the establishment of hypertension in the model. After the experiment, heart and kidney tissues were collected and subjected to H&E staining. Excluding the animal that died due to anesthesia-related complications, histological evaluation was performed on all remaining rats (n=4). In the heart, thickening of the left ventricular wall was noted compared to normotensive controls, consistent with cardiac hypertrophy secondary to elevated blood pressure (Figure S5C). In the kidneys, destruction of some glomeruli was observed, indicating renal damage likely caused by sustained hypertension (Figure S5D). These findings are in line with previously reported characteristics of Angiotensin-II induced hypertension models [3].

Supplementary Note 3. Controller tuning through simulation

The engineering characteristics of the model were evaluated using the transition matrix (Figure S8A). All four eigenvalues of the identified model were located within the unit circle on the complex plane, indicating that the system is asymptotically stable. Specifically, the eigenvalue on the positive real axis converged monotonically without sign changes, while the one on the negative real axis exhibited alternating convergence with sign changes but decayed rapidly with small magnitude. The remaining two eigenvalues formed a complex conjugate pair, resulting in damped oscillations with gradually decreasing amplitude over time. These findings demonstrate that all eigenvalues lie within the stable region and exhibit convergent behavior,

indicating that the model is dynamically stable.

Additionally, the dynamics matrix of the IML was confirmed to be full-rank, verifying the controllability of the system (Supplementary Note 4). This ensures that the system can be guided from an initial state to a desired target state using an appropriate controller. The system was also confirmed to be observable, meaning that the internal states can be estimated using a properly designed observer. These characteristics provide a foundation for continuously monitoring system states and regulating them to achieve the desired output.

Subsequently, the controller and observer were designed separately based on the separation principle (Figure S8B). Controller parameter tuning was a critical step in determining the convergence rates of the input and output. Parameters were adjusted through simulations based on the previously fitted model. The weighting terms in the cost function were intentionally set to limit the stimulation frequency to below 30 Hz for safety and to achieve slow convergence (Figure S8C). The tuned Kalman filter reliably estimated the system states despite the presence of noise (Figure S8D).

Supplementary Note 4. Parameters of CL system

The parameters of IML dynamic and LQG were set in 4 order.

LSSM:

$$\dot{x} = Ax + Bu \qquad (1)$$

$$y = Cx + Du \qquad (2)$$

$$A = \begin{bmatrix} -0.2018 & 0.8000 & -0.4485 & -0.1593 \\ -0.1044 & 0.5163 & 0.8649 & 0.1369 \\ 0.0244 & 0.2443 & -0.0629 & 0.4012 \\ -0.0149 & 0.0440 & -0.1267 & -0.3213 \end{bmatrix}$$

$$B = \begin{bmatrix} -1.9572 \\ 0.0612 \\ 0.0414 \\ -0.0107 \end{bmatrix}$$

$$C = \begin{bmatrix} -0.9732 & -0.2235 & 0.0137 & 0.0308 \end{bmatrix}$$

$$D = [0];$$

LQR Controller:

$$K = \begin{bmatrix} -0.0020 & -0.0103 & 0.0047 & 0.0017 & -0.0032 \end{bmatrix}$$

$$Q = [1.1023], \quad R = \begin{bmatrix} 110780 & 0 \\ 0 & 980126 \end{bmatrix}$$

Kalman gain:

$$L = \begin{bmatrix} 0.0242 \\ 0.0084 \\ -0.0016 \\ 0.0013 \end{bmatrix},$$

Supplementary Reference

1. Wichmann, T., *A digital averaging method for removal of stimulus artifacts in neurophysiologic experiments.* Journal of Neuroscience Methods, 2000. **98**(1): p. 57-62.
2. Yang, Y., et al., *Modelling and prediction of the dynamic responses of large-scale brain networks during direct electrical stimulation*, in *Nat Biomed Eng*. 2021. p. 324-345.
3. Crowley, S.D., et al., *Angiotensin II causes hypertension and cardiac hypertrophy through its receptors in the kidney.* Proc Natl Acad Sci U S A, 2006. **103**(47): p. 17985-90.

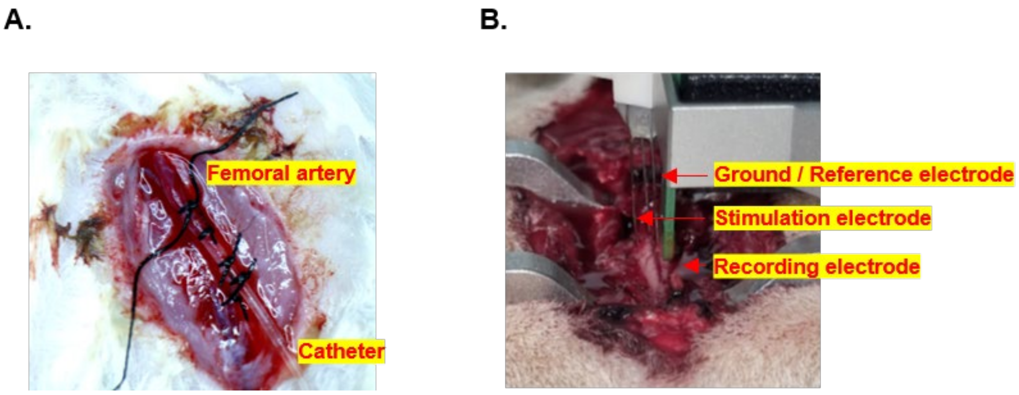

Figure S1. Experiment setup.

A. Femoral artery catheterization. **B.** Electrodes placed on the spinal cord.

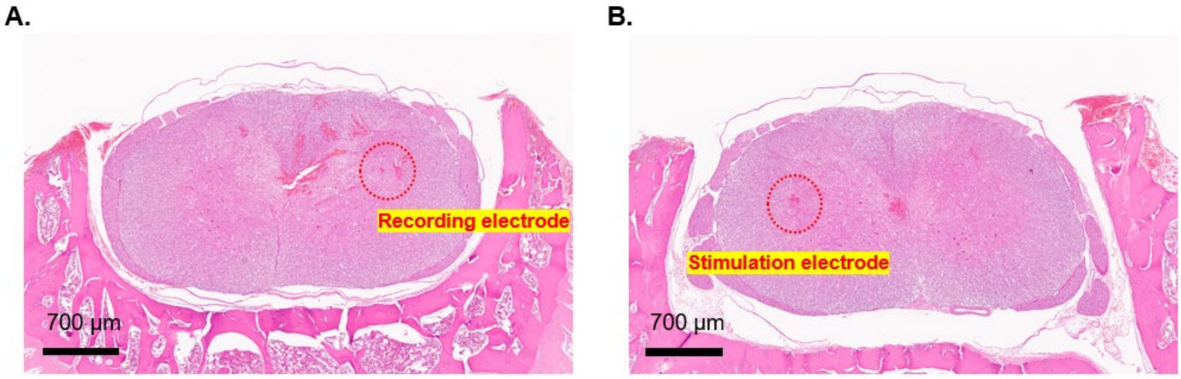

Figure S2. Histological results of spinal cord cross-sections for electrode placement verification

A. Cross-sectional view of the left T13 IML. The stimulation electrode location is indicated by a red dashed circle. **B.** Cross-sectional view of the right L1 IML. The recording electrode location is indicated by a red dashed circle.

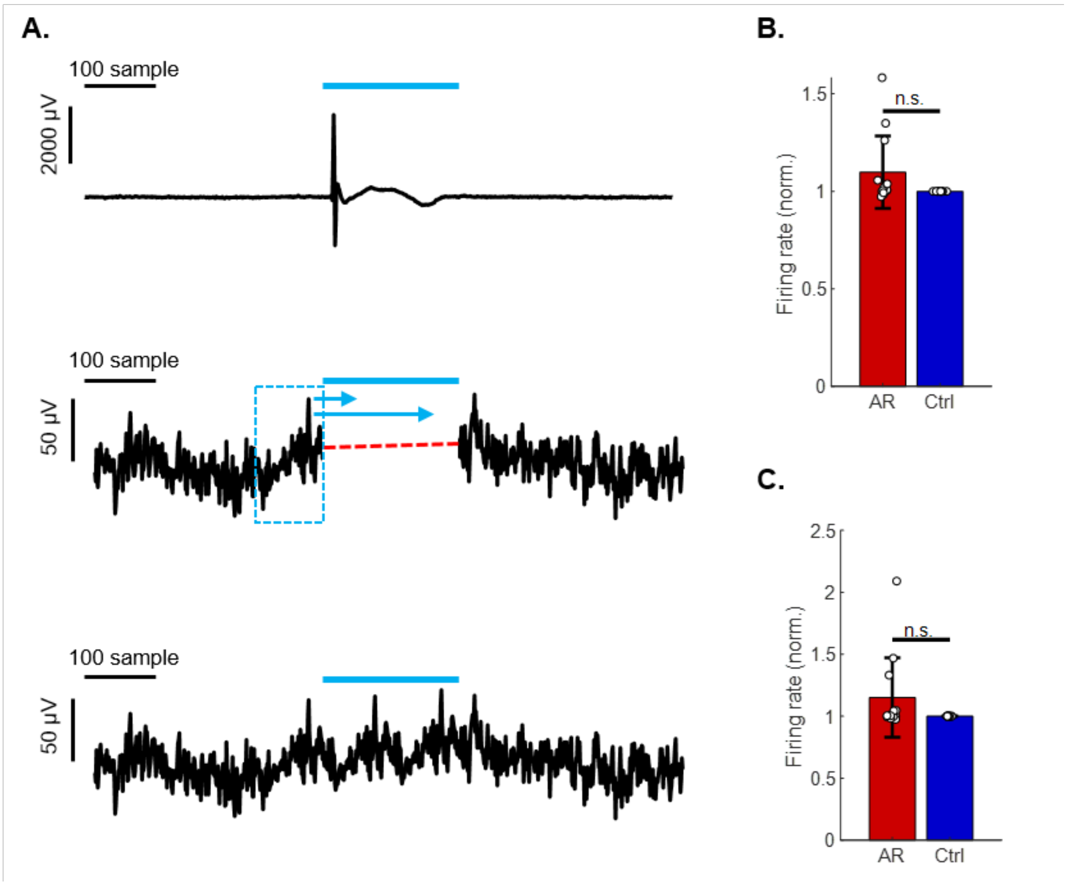

Figure S3. Stimulation artifact rejection method and distortion rate evaluation

A. An example channel shows the procedure for stimulation artifact rejection. A 192-sample segment of the raw signal (blue line) was interpolated using the signal preceding the artifact. **B.** Distortion caused by 30 Hz stimulation. **C.** Distortion caused by 40 Hz stimulation. For panel B and C, group differences were assessed using the two-sided Wilcoxon signed-rank test (n=12, n.s: not significant, *: $p < 0.01$, **: $p < 0.01$, ***: $p<0.001$).

Abbreviations: AR: Artifact-removed signal; Ctrl: Control

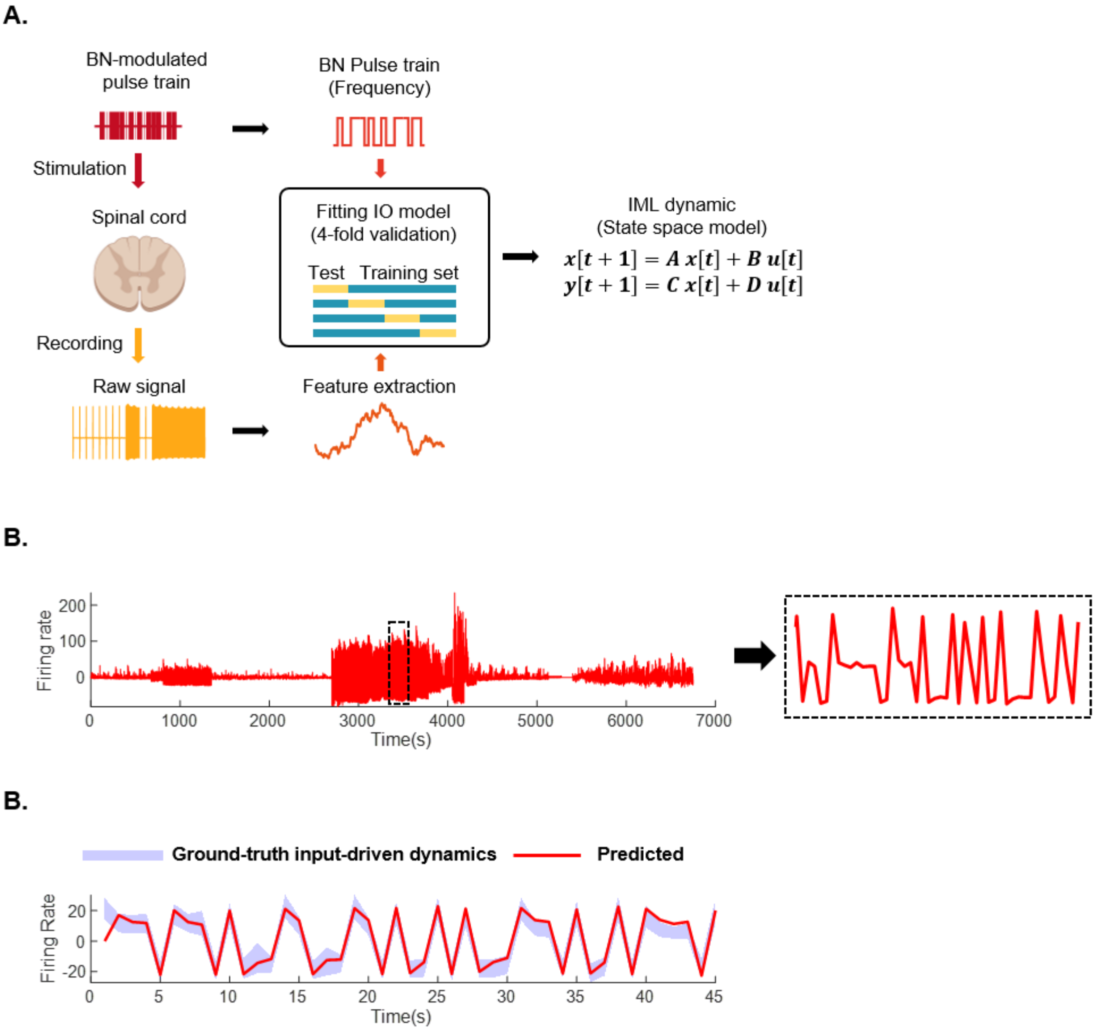

Figure S4. Details of input-output data-driven model.

A. Identification progress of input-output data-driven model. **B.** Representative example of train data. **C.** Representative example of output prediction performance. The predicted value (red line) closely matches the ground-truth input-driven dynamics (blue area).

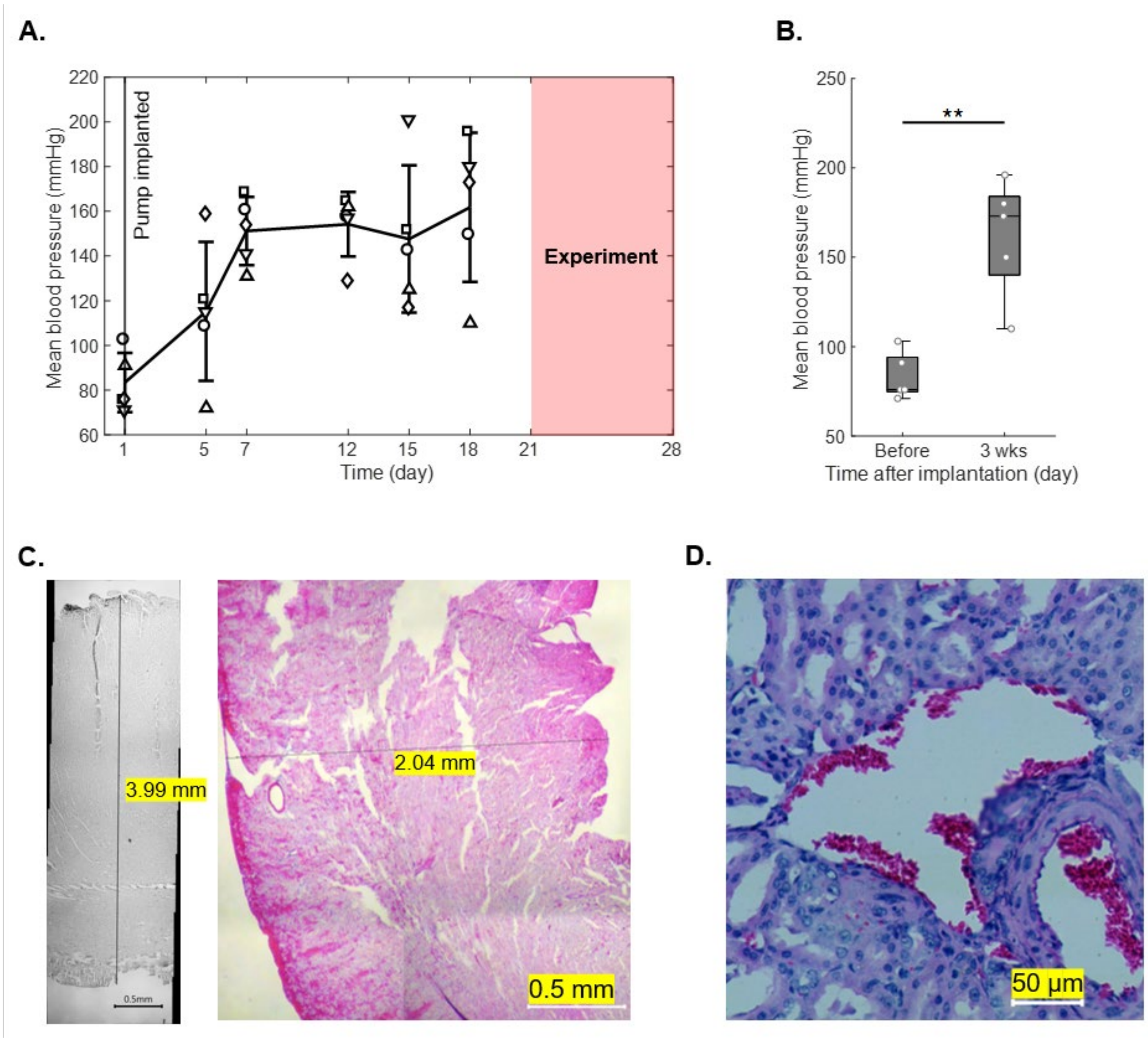

Figure S5. Validation of Angiotensin-II induced hypertension model.

A. Blood pressure measurements obtained using the tail-cuff method following pump implantation. Each individual is represented by a distinct marker. Considering the pump's lifespan, no measurements were taken between days 21 and 28 after implantation. **B.** Comparison of mean blood pressure before and three weeks after pump implantation. Statistical significance was evaluated using the two-sided Wilcoxon signed-rank test (n.s: not significant, *: $p < 0.01$, **: $p < 0.01$, ***: $p<0.001$). **C.** Representative image of left ventricular anterior wall thickness. Cardiac hypertrophy was observed in the hypertensive model (left) compared to the normotensive control (right). **D.** Histological analysis of the kidney in the hypertensive

model. Destruction of some glomeruli indicated renal damage induced by hypertension.

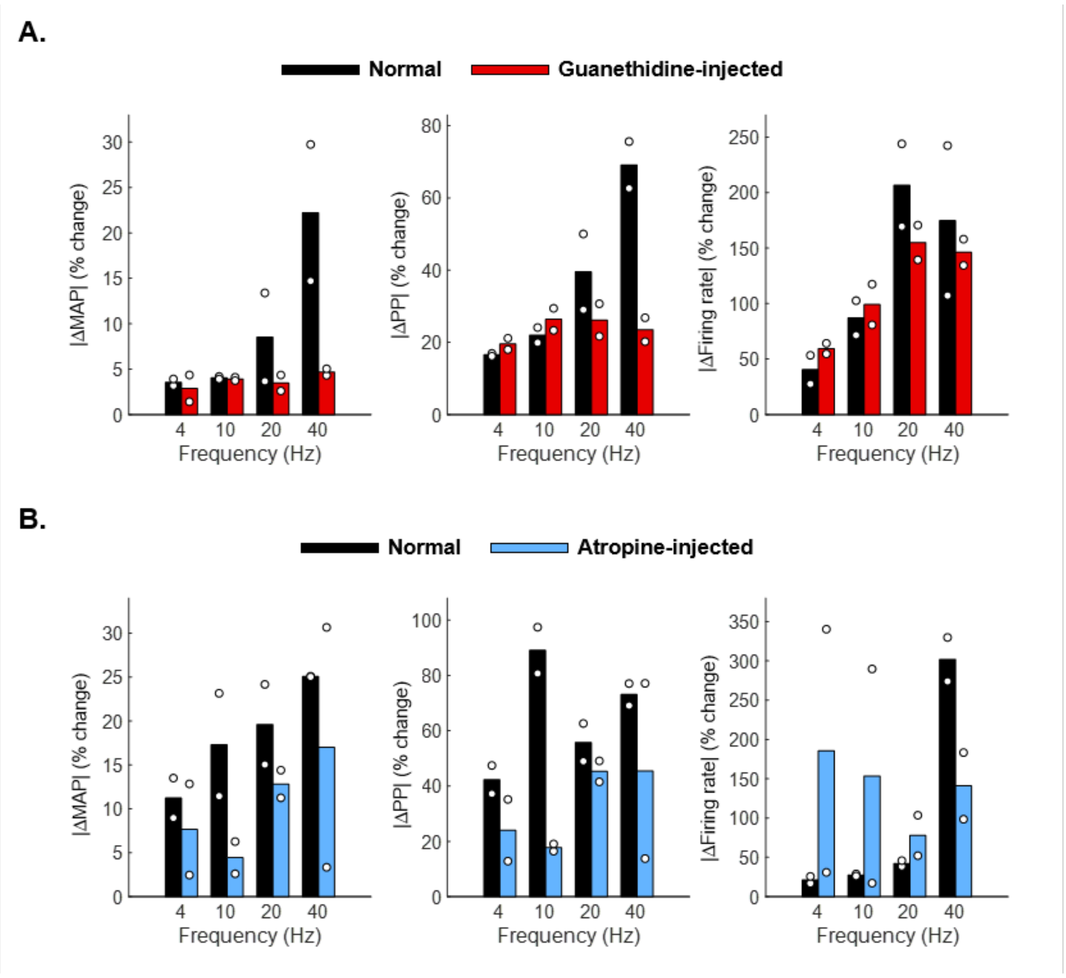

Figure S6. Effects of direct intermediolateral nucleus stimulation on cardiovascular and neural responses following chemical autonomic denervation.

A. Absolute changes in mean arterial pressure, heart rate, pulse pressure, and IML firing rate in response to direct IML stimulation, relative to baseline, under normal conditions (black) and following administration of the sympathetic blocker guanethidine (red) (from 1 rat, n = 2). **B.** Absolute changes in mean arterial pressure, heart rate, pulse pressure, and IML firing rate in response to direct IML stimulation, relative to baseline, under normal conditions (black) and following administration of the parasympathetic blocker atropine(blue) (from 1 rat, n = 2).

Abbreviations: MAP: mean arterial pressure, HR: heart rate, PP: pulse pressure, IML: intermediolateral nucleus

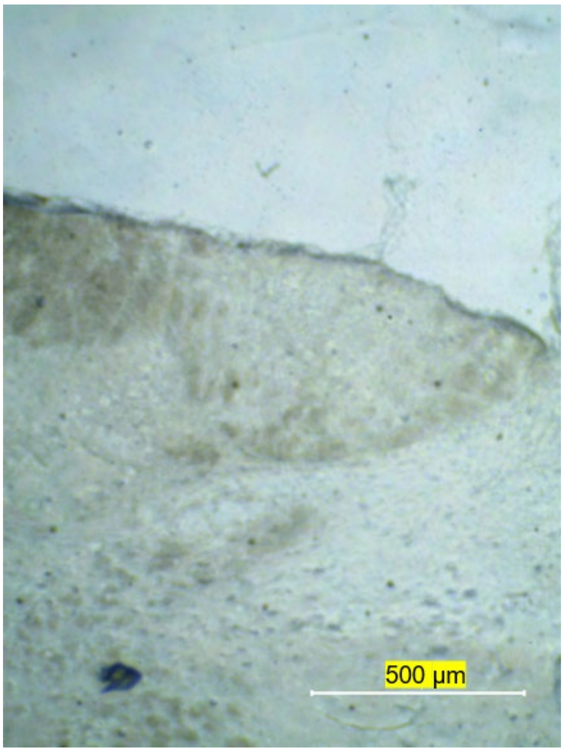

Figure S7. c-Fos activation in the nucleus tractus solitarius following direct intermediolateral nucleus Stimulation.

c-Fos expression level in the nucleus tractus solitarius following direct intermediolateral nucleus Stimulation.

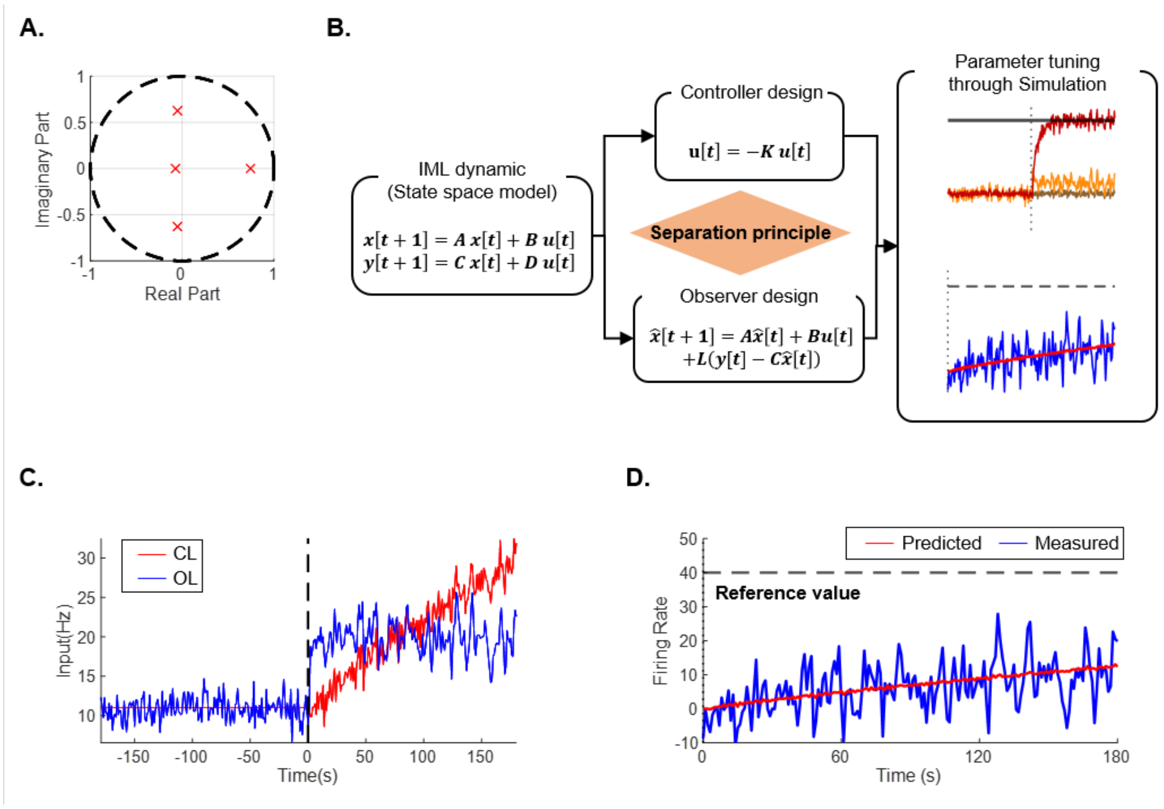

Figure S8. Tuning of closed-loop algorithm.

A. Transition matrix of identified linear state space model. **B.** Parameter Tuning progress based on the separation principle. **C.** Input simulation results over 180 seconds with the tuned input weight. **D.** Output signals including the noise-corrupted measurements (blue line) and the estimated values using the Kalman filter (red line).

Supplementary Table 1. Statistical result

Figure	Panel	Statistical test used	Sample definition	Descriptive statics shown	P value
2	C	Kruskal–Wallis test with post hoc Dunn–Šidák correction	n = 7	Box plots with whiskers	p = 0.002 for Kruskal-Wallis test<br><br>Post-hoc test results<br>P = 0.4134, 0.2205, < 0.001,<br>0.9997, 0.1910, 0.3693<br>for 4 Hz vs 10 Hz; 4 Hz vs 20 Hz; 4 Hz vs 40 Hz,<br>10 Hz vs 20 Hz; 10 Hz vs 20 Hz, 20 Hz vs 40 Hz each
	D	Kruskal–Wallis test with post hoc Dunn–Šidák correction	n = 7	Box plots with whiskers	P < 0.001 for Kruskal-Wallis test<br><br>Post-hoc test results<br>P = 0.9434, 0.2205, < 0.001,<br>0.8105, 0.0097, 0.2532

					for 4 Hz vs 10 Hz; 4 Hz vs 20 Hz; 4 Hz vs 40 Hz,<br>10 Hz vs 20 Hz; 10 Hz vs 20 Hz, 20 Hz vs 40 Hz each
	G	Kruskal–Wallis test with post hoc Dunn–Šidák correction	n = 7	Box plots with whiskers	p = 0.0041 for Kruskal-Wallis test<br><br>Post-hoc test results<br>P = 0.6153, 0.7151, 0.3941,<br>1.0000, 0.0082, 0.0130<br>for 4 Hz vs 10 Hz; 4 Hz vs 20 Hz; 4 Hz vs 40 Hz,<br>10 Hz vs 20 Hz; 10 Hz vs 20 Hz, 20 Hz vs 40 Hz each
	H	Kruskal–Wallis test with post hoc Dunn–Šidák correction	n = 7	Box plots with whiskers	p = 0.0174 for Kruskal-Wallis test<br><br>Post-hoc test results<br>P = 0.7694,0.4597, 0.0097<br>0.9988, 0.2889, 0.5812<br>for 4 Hz vs 10 Hz; 4 Hz vs 20 Hz; 4 Hz vs 40 Hz,

					10 Hz vs 20 Hz; 10 Hz vs 20 Hz, 20 Hz vs 40 Hz each
	J	Kruskal–Wallis test with post hoc Dunn–Šidák correction	n = 7	Box plots with whiskers	p = 0.0068 for Kruskal-Wallis test<br><br>Post-hoc test results<br>P = 0.7253, 0.8956, 0.3277, 0.9999, 0.0097, 0.0252<br>for 4 Hz vs 10 Hz; 4 Hz vs 20 Hz; 4 Hz vs 40 Hz, 10 Hz vs 20 Hz; 10 Hz vs 20 Hz, 20 Hz vs 40 Hz each
3	B left	Kruskal–Wallis test with post hoc Dunn–Šidák correction	n = 17, from 6 rats	Box plots with whiskers	P <0.001 for Kruskal-Wallis test,<br><br>Post-hoc test results<br>P < 0.001, 0.9485, <0.001<br>for pre vs stim, pre vs post, stim vs post
	B right	Kruskal–Wallis test	n = 17, from 6 rats	Box plots with	P = 0.0221 for Kruskal-Wallis test,

		with post hoc Dunn–Šidák correction		whiskers	Post-hoc test results<br>P = 0.5043, 0.0350, 0.0173<br>for pre vs stim, pre vs post, stim vs post
	C	Two-sided Wilcoxon signed-rank test.	n = 17, from 6 rats	Full dataset & error bars are mean ±SD	P = 0.0042
			n = 6	Full dataset & error bars are mean ±SD	P = 0.0313
	D	Two-sided Wilcoxon signed-rank test.	n = 17, from 6 rats	Full dataset & error bars are mean ±SD Full	P = 0.0191

				dataset & error bars are mean ±SD	
			n = 6	Full dataset & error bars are mean ±SD	P = 0.0313
	E	Two-sided Wilcoxon signed-rank test.	n = 17, from 6 rats	Full dataset & error bars are mean ±SD	P < 0.001
			n = 6	Full dataset & error bars are mean ±SD	P = 0.0313

	F	Two-sided Wilcoxon signed-rank test.	n = 17, from 6 rats	Box plots with whiskers	P = 0.0468
			n = 6	Full dataset & error bars are mean ±SD	P = 0.8438
	H	Two-sided Wilcoxon signed-rank test.	n = 17, from 6 rats	Full dataset & error bars are mean ±SD	P = 0.0245
			n = 6	Full dataset & error bars are mean ±SD	P = 0.6250

	I left	Two-sided Wilcoxon signed-rank test	n = 17, from 6 rats	Box plots with whiskers	P = 0.4348
	I right		n = 17, from 6 rats	Box plots with whiskers	P = 0.0312
4	C	Two-sided Wilcoxon signed-rank test.	n = 15, from 5 rats, 3 trials for each.	Box plots with whiskers	P = 0.0134
5	B left	Kruskal–Wallis test with post hoc Dunn–Šidák correction	n = 12	Box plots with whiskers	P < 0.0001 for Kruskal-Wallis test,<br><br>Post-hoc test results<br>P < 0.001, 0.1053, 0.0506<br>for pre vs stim, pre vs post, stim vs post

	B right	Kruskal–Wallis test with post hoc Dunn–Šidák correction	n = 12	Box plots with whiskers	$P < 0.001$ for Kruskal-Wallis test,<br><br>Post-hoc test results<br>$P < 0.001$, 0.4260, 0.0533<br>for pre vs stim, pre vs post, stim vs post
	C left	Two-sided Wilcoxon signed-rank test.	n = 12, from 4 rats, 3 trials for each.	Full dataset & error bars are mean ±SD	$P < 0.001$
	C right		n = 4	Full dataset & error bars are mean ±SD	$P = 0.1250$
	D left	Two-sided Wilcoxon	n = 12, from 4 rats, 3 trials	Full dataset & error bars	$P = 0.0068$

		signed-rank test.	for each.	are mean ±SD	
	D right		n = 4	Full dataset & error bars are mean ±SD	P = 0.1250
	E left	Two-sided Wilcoxon signed-rank test.	n = 12, from 4 rats, 3 trials for each.	Box plots with whiskers	P = 0.0098
	E right		n = 4	Box plots with whiskers	P = 0.1250
	F left	Two-sided Wilcoxon signed-rank	n = 12, from 4 rats, 3 trials for each.	Full dataset & error bars are mean	P = 0.0029

		test.		±SD	
	F right		n = 4	Full dataset & error bars are mean ±SD	P = 0.1250
	G left	Two-sided Wilcoxon signed-rank test.	n = 12, from 4 rats, 3 trials for each.	Box plots with whiskers	P = 0.0400
	G right		n = 4	Box plots with whiskers	P = 0.2500
	I left	Two-sided Wilcoxon signed-rank test	n = 12, from 4 rats	Box plots with whiskers	P = 0.5693
	I right		n = 12, from 4	Box plots	P = 0.0210

			rats	with whiskers	
S3	B	Two-sided Wilcoxon signed-rank test.	n = 12, from 6 rats.	Full dataset & error bars are mean ±SD	0.0575
	C	Two-sided Wilcoxon signed-rank test.	n = 12, from 6 rats.	Full dataset & error bars are mean ±SD	P = 0.0599
S8	B	Two-sided Wilcoxon signed-rank test.	n = 5	Box plots with whiskers	P = 0.0088
